# Sex Differences in Metabolite–Immune Circuits of Neuroinflammation

**DOI:** 10.1111/imr.70114

**Published:** 2026-03-09

**Authors:** Priyanka Saminathan, Maija Corey, Alicia Gibbons, Mahati Rayadurgam, Neha Reddy, Pavithra Ramesh, Sonia Sharma

**Affiliations:** ^1^ La Jolla Institute for Immunology La Jolla California USA; ^2^ University of California San Diego La Jolla California USA; ^3^ Laboratory for Inflammatory Immune Metabolism Center for Integrative Medical Sciences, RIKEN Yokohama Japan

**Keywords:** bio‐active lipids, immunometabolism, microglia, neuroinflammation, purine metabolism, sex differences

## Abstract

Sex is a fundamental yet underexplored determinant of human neuroinflammation. Across autoimmune, neurodegenerative, and post‐infectious neurological syndromes, males and females exhibit consistent differences in disease vulnerability, progression, and immune tone. While sex hormones and chromosomes strongly shape immune development and function in health and disease, they do not fully explain the magnitude or disease‐specific patterns of these disparities, nor do they provide sufficient mechanistic information for developing novel therapeutics. Emerging evidence suggests that sex‐defining factors interact with age and environment to shape downstream metabolite–immune circuits, networks in which metabolic enzymes, metabolites, and immune cells tune inflammatory set points. Pathways spanning purine metabolism, glycolytic remodeling, lipid sensing, mitochondrial stress, and nucleic‐acid sensing can recalibrate microglial activation thresholds, T‐cell cytokine programs, innate type I interferon antiviral responses, and shape overall CNS resilience in a sex‐dependent manner. Here, we synthesize mechanistic and human systems‐level studies to propose an integrated framework in which sex‐biased immunometabolism serves as a mechanistic bridge between biological sex and neuroimmune disease risk, progression, and responses to injury. We highlight key knowledge gaps and discuss how targeting metabolite–immune pathways may enable sex‐informed biomarkers and therapeutic strategies in neuroinflammatory disease.

## 1 Introduction

Neuroinflammation is a defining feature of many neurological and neurodegenerative diseases, playing a central role in reprogramming cellular states and shaping brain homeostasis, tissue injury, repair and long‐term clinical outcomes. Within the central nervous system (CNS), tissue‐resident and infiltrating immune cells act as both drivers and resolvers of inflammation, orchestrating the local immunological landscape, as demonstrated in mouse models and supported by human disease studies [1, 2]. Across neurological disorders as diverse as multiple sclerosis (MS) [3], amyotrophic lateral sclerosis (ALS) [4, 5], Alzheimer's disease (AD) [6, 7], Parkinson's disease (PD) [8], autoimmune encephalitis [9, 10], stroke [11, 12], traumatic brain injury [13] and post‐infectious or treatment‐related syndromes such as long COVID [14, 15], the CNS exhibits striking sexually dimorphic patterns of disease susceptibility, progression and inflammatory tone. Females disproportionately develop MS, AD, autoimmune encephalitis and many chronic pain and fatigue syndromes, whereas males more frequently experience severe disease progression in PD, ALS and ischemic injury. Collectively, these disparities point to fundamental sex‐specific differences in brain immunology shaped by sex chromosomal complement, hormones and environmental influences. How these upstream factors are translated into durable differences in neuroimmune function remains incompletely understood, pointing to the existence of downstream integrative pathways that bridge sex, environment and inflammation.

Despite robust evidence for sex bias in neuroimmune disorders, the mechanistic basis underlying these differences remains incompletely understood. Studies focused on the effects of sex chromosomes and gonadal hormones in experimental animal models have provided important insights into immune calibration and microglial maturation [16, 17], yet they do not fully account for the magnitude, persistence or disease‐specific patterns of sexual dimorphism observed across the CNS in human neurological disease [7, 18, 19]. At a mechanistic level, these upstream factors converge on distinct metabolic–immune circuits that regulate neuroimmune function across health and disease. Functional studies in both animal models and human immune cells show that purine and pyrimidine metabolism, lipid pathways and mitochondrial stress responses are all directly linked to immune cell and signaling modules as critical regulatory nodes through which sex differences can shape neuroinflammation [20, 21, 22]. This framework is particularly compelling given the CNS's exceptional energetic demand, limited metabolic flexibility and reliance on finely tuned interactions among microglia, astrocytes, neurons and infiltrating immune cells. Perturbations in nucleoside/nucleotide pools, lipid intermediates and other key metabolites can disproportionately alter microglial activation thresholds, antiviral responses, phagocytic behavior and synaptic maintenance, thereby destabilizing neuroimmune homeostasis [23, 24, 25].

Recent mechanistic studies, including work from our laboratory, define how bioactive metabolites and their associated regulatory enzymes intersect with immune cell signaling pathways involved in defense responses against pathogens [26, 27, 28]. Loss‐of‐function studies of the tumor‐suppressor kinase Death‐Associated Protein Kinase 3 (DAPK3), a metabolic stress–responsive regulator of cell death and autophagy expressed in neurons, endothelial cells and myeloid‐lineage cells including microglia, identified DAPK3 as an essential upstream activator of innate immune cyclic GMP–AMP synthase (cGAS)–Stimulator of Interferon Genes (STING) signaling in human and mouse cells [27]. In this pathway, cGAS senses cytosolic DNA danger signals of microbial origin or generated by genomic or mitochondrial stress, triggering STING‐dependent signaling to generate type I interferon and its associated immune response. Multiple studies, mostly conducted in mouse models, now implicate cGAS–STING signaling in CNS health and disease, showing that microbial, mitochondrial or nuclear DNA stress activates STING in microglia and other CNS‐resident cells during aging, stroke, traumatic injury and Alzheimer's disease, where it drives type I interferon responses associated with synapse loss, neuroinflammation and neurodegenerative progression [29, 30]. Mechanistically, DAPK3 is essential for STING activation, and directly interacts with STING and promotes its K63‐linked ubiquitination, enabling downstream IFNβ production and associated immune responses [27].

Functional characterization of the metabolic enzymes that regulate systemic purine nucleoside homeostasis revealed that the adenosine deaminases (i.e., ADA1 and ADA2) control a purine‐regulated innate immune circuit that determines the magnitude of the IFNβ immune‐inflammatory response [28, 31, 32]. In human cells, ADA deficiency and/or accumulation of deoxyadenosine and deoxyinosine metabolites perturb cellular methionine–S‐adenosylmethionine (SAM) metabolism, leading to decreased SAM trans‐methylation potential and epigenetic derepression of human endogenous retroviral elements (HERVs) encoded in cellular genome [28]. HERV‐derived transcripts engage innate sensors of double‐stranded RNA, including Toll‐like receptor 3 (TLR3) and Retinoic Acid Inducible gene I (RIG‐I), thereby priming basal IFNβ expression [28]. In humans, germline ADA2 deficiency (DADA2) manifests clinically as a syndrome of chronic vascular inflammation and stroke, implicating dysregulated IFNβ signaling in this disease pathology [31, 33]. Human cohort studies have demonstrated that ADA enzymatic activity is sexually dimorphic, with male subjects exhibiting higher ADA2 activity than female counterparts [34, 35]. Across groups of healthy individuals and patients with COVID‐19, lower ADA activity in females associated with enhanced expression of type I IFN–driven antiviral gene signatures in the lung [36, 37], situating purine metabolism within a broader framework of sex‐ and tissue‐specific immune regulation [34].

Additional work links mitochondrial purine metabolism to innate immune activation and neurological disease. Disruption of deoxyguanosine kinase (DGUOK), the mitochondrial enzyme responsible for phosphorylating deoxyadenosine to its monophosphate derivative, in human hepatocytes induces a robust IFNβ and interferon‐stimulated gene (ISG) program [35]. Given that DGUOK deficiency causes a rare disorder characterized by hepatic and neurological dysfunction, these findings implicate a previously underappreciated immunometabolic axis with direct relevance to neurological disease. Nucleoside metabolism also shapes adaptive immunity. A functional genetic screen in endogenous human Th1 memory CD4^+^ T cells, the long‐lived helper T cells that retain immunological memory of intracellular bacterial infections such as *Mycobacterium tuberculosis* , identified isochorismatase domain‐containing protein 1 (ISOC1) as a key regulator linking pyrimidine metabolism to IFNγ and IL‐17 production [35, 36]. Disruption of ISOC1 impaired effector cytokine output, which was rescued by supplementation with extracellular pyrimidines, highlighting direct metabolic control of human T‐cell effector function.

Collectively, these studies support a model in which metabolic enzymes (ADA1, ADA2, ISOC1, DGUOK) and innate immune sensors (TLR3, RIG‐I, and STING via DAPK3) operate as integrated circuits that tune the amplitude and chronicity of IFN‐driven inflammation in a tissue‐ and sex‐dependent manner. When considered alongside growing evidence that innate IFN signaling and purine metabolism shape microglial states and CNS injury responses [28, 29, 30, 32], these findings suggest that sex differences in neuroinflammation may arise, in part, from differential wiring of metabolic–immune pathways within microglia and other cells at the neuroimmune interface. Despite these advances, no unified framework currently synthesizes how sex, metabolism, and neuroimmune compartments converge to govern neuroinflammatory disease risk, progression, and burden. The field lacks comprehensive, sex‐stratified neuroimmune maps integrating transcriptomic, lipidomic, and metabolomic data with functional states of CNS‐resident and infiltrating immune populations across developmental stages and hormonal contexts. In this review, we outline current knowledge of sex differences in neuroinflammation, highlight emerging metabolic–immune pathways as key determinants of sexually dimorphic CNS inflammation, and integrate recent mechanistic studies as examples of this conceptual framework. We conclude by identifying unmet needs in experimental models, multi‐omic approaches, and therapeutic strategies aimed at targeting metabolic–immune circuits in a sex‐informed manner.

## 2 Sex Differences in Neuroinflammatory Diseases

Significant sex differences are extensively described across neurocognitive and neurodegenerative diseases in humans (summarized in Table 1). Females exhibit a substantially higher risk of autoimmune CNS disorders, most notably multiple sclerosis (MS). Large epidemiological studies demonstrate that MS incidence is approximately two to threefold higher in females than in males [38, 39]. Although MS is more prevalent in females, male patients tend to experience a more aggressive disease trajectory, characterized by faster disability accumulation and earlier conversion to secondary progressive MS [40]. Autoimmune encephalitis similarly displays pronounced sex bias. Anti–NMDA receptor encephalitis occurs predominantly in females, with approximately 80% of cases occurring in females and a female‐to‐male ratio of roughly 4:1 [40, 41]. While this disparity is partly attributable to ovarian teratomas, which may contain neural tissue expressing NMDA receptor subunits, it is also observed in teratoma‐negative females, indicating additional sex‐specific biological contributors beyond tumor‐associated immunity.

**TABLE 1:** Summary of sex differences in human neurological diseases.

Disease	Sex bias in prevalence	Sex bias in presentation	Key publications and reviews
Alzheimer's disease (AD)	Higher prevalence in women (~2:1 F:M)	Women have greater lifetime risk; faster cognitive decline reported in several cohorts	Castro‐Aldrete L et al. PMID:37065460, Rosende‐Roca M et al. PMID:39844303, Lin KA et al. PMID:26451386
Amyotrophic lateral sclerosis (ALS)	Slight male predominance (~1.5:1 M:F)	Men present younger with more spinal‐onset disease; shorter survival in several cohorts	Fontana A et al. PMID:33630135, Manjaly ZR et al. PMID:20225930, Grassano M et al. PMID:38568048, McCombe PA, et al. PMID: 21195356
Autoimmune encephalitis (AE)	Female‐predominant overall (~4:1 F:M, but observed sex bias is disease subtype‐specific)	Anti‐NMDA receptor encephalitis has a female: male ratio of roughly 4:1. By contrast, other AE subtypes (e.g., LGI1 or CASPR2 antibody encephalitis) show male predominance (often older men)	Dalmau J et al. PMID:31326280, Shan et al. PMID: 33679765, Gold SM et al. PMID: 30361800
Multiple sclerosis (MS)	~2–3:1 female‐to‐male prevalence	Males show faster progression and worse disability accumulation	Riley Bove et al. PMID:23608496, Marrie RA et al. PMID: 33239364, Bove et al. PMID: 23608496
Parkinson's disease (PD)	Higher incidence in men (~3–4:1 M:F)	Women have later onset and slower progression; estrogen implicated as protective (evidence mixed)	Cerri S et al. PMID:31282427, Zirra A et al. PMID:36699001
Stroke	Higher mid‐life incidence in men	Women experience stroke at older ages and have poorer post‐stroke functional outcomes	Vyas MV et al. PMID:33493057, Yun SM et al. PMID:36786321

Sex bias is also evident in neurodegenerative diseases. Among the approximately 5.1 million Americans over the age of 65 living with Alzheimer's disease (AD), nearly 3.2 million are females [42]. Although increased female longevity may contribute to the imbalance, sex‐specific immune responses, microglial activation states, and interactions with APOE‐ε4 are increasingly recognized as additional biological factors [43]. In contrast, Parkinson's disease (PD) is significantly more prevalent in males, with meta‐analyses reporting a male‐to‐female incidence of approximately 1.5–2.0, with some cohorts approaching ratios of 3–4 across specific age groups [44]. In addition to higher male incidence, females with PD often exhibit slower motor progression, including reduced rates of UPDRS‐III decline and better preservation of dopaminergic imaging markers [45]. Males not only develop amyotrophic lateral sclerosis (ALS) more frequently than females, with a male‐to‐female ratio of approximately 1.3–1.6, but also show shorter survival, driven largely by more rapid respiratory decline and faster deterioration in forced vital capacity [46, 47]. Conversely, several studies report that females with ALS may experience more rapid decline in limb motor function [48]. These observations underscore that sex differences in ALS are domain‐specific rather than uniformly favoring one sex.

Acute neurological disorders also show marked sex‐specific patterns. Stroke incidence is higher in males at younger ages, particularly between 55 and 75 years, with males demonstrating a 33%–50% higher age‐adjusted risk in these strata [49, 50]. However, females, especially postmenopausal females, experience worse functional recovery, including higher 90‐day modified Rankin Scale scores, greater long‐term disability and lower rates of functional independence following ischemic stroke [51, 52]. These differences persist after adjustment for age and comorbidities, suggesting biological contributors beyond social or healthcare‐access factors. Together, epidemiological and clinical patterns indicate that sex differences in neuroinflammatory disease are robust, domain‐specific and mechanistically heterogeneous, reflecting the involvement of interactions between immune regulation, hormonal milieu, metabolic state, and genetic susceptibility. Epidemiological data thus clearly establish sex as a major determinant of disease vulnerability and outcome, motivating a deeper examination of the biological mechanisms underlying these disparities.

An important but largely underexplored question is whether males and females respond differently to disease therapies. Sex‐stratified analyses are still limited in clinical trial data, constraining definitive conclusions. In MS, males generally exhibit worse prognosis; however, available data suggest that first‐line disease‐modifying therapies such as IFNβ supplementation and glatiramer acetate show broadly comparable efficacy between sexes, although rigorous, adequately powered comparisons are still lacking [53]. Sex differences may influence treatment tolerability and optimal dosing through pharmacokinetic and metabolic effects. In PD, females experience higher rates of dyskensias after first‐line levodopa therapy, potentially reflecting differences in drug metabolism or body composition, whereas males may derive less benefit from certain interventions such as deep brain stimulation [54]. After stroke, females are less likely to receive acute thrombolysis and often demonstrate poorer rehabilitation outcomes [55, 56]. Despite these observations, sex is rarely incorporated into therapeutic decision‐making, representing a significant gap in clinical practice. Incorporating sex‐specific immune and metabolic differences into trial design and treatment strategies may improve personalization of neuroinflammatory disease management.

Despite growing recognition of sex differences in neuroinflammation, mechanistic understanding remains incomplete. In particular, how sex, metabolism, and immune signaling intersect within the CNS is unresolved. While robust sex differences in peripheral immune responses are well documented, comparatively few studies have examined sex‐specific neuroimmune interactions within the brain. As a result, key mechanistic questions remain unanswered. For example, how do female and male microglia differ in their metabolic reprogramming during neurodegeneration, and do these differences influence disease trajectory or severity? How do sex hormones interface with metabolic regulators and innate immune sensors in the aging brain? Converging hormonal, genetic, metabolic, and immune pathways are likely to act in combination rather than isolation, yet integrative studies addressing their interaction are scarce. Emerging areas such as immunometabolism and epigenetic regulation in the context of sex differences remain particularly underexplored in neuroinflammatory diseases. Most observations of sex bias derived from clinical cohorts or animal models do not identify causal mechanisms. For instance, although females develop MS more frequently than males, the drivers of this bias within the CNS remain poorly defined beyond differences in circulating immune populations.

Addressing these gaps will require coordinated approaches, including transcriptomic, epigenetic, and metabolic profiling of male and female patient‐derived CNS cells; systematic investigation of sex chromosome effects on brain‐resident immune cell development; and evaluation of sex‐specific responses to emerging immunotherapies. Elucidating how sex, immune signaling, and metabolism converge within the CNS will not only clarify disease pathogenesis but also inform the development of sex‐informed therapeutic strategies. Sex differences therefore represent a fundamental dimension of neuroinflammatory disease biology, and resolving these gaps—particularly at the level of metabolic and innate immune regulation—will be essential for advancing neuroimmunology and improving patient care.

### 2.1 Biological Contributors to Sex Differences in Neuroinflammation

Sex differences in neuroinflammation arise from a complex interplay of biological factors, most prominently sex hormones and sex chromosome complement. Together, these factors contribute to intrinsic immune response biases between males and females and shape neuroimmune function across the lifespan. Below, we consider each of these contributors.

#### 2.1.1 Hormonal Influences on Neuroinflammation

Sex steroid hormones exert profound effects on immune responses within both the CNS and the periphery. Estrogens (including estradiol), progesterone, and androgens signal through nuclear and membrane‐associated receptors (e.g., GPER1) expressed on microglia, astrocytes, neurons, dendritic cells, T cells and B cells [57, 58, 59]. Activation of these receptors modulates cytokine production, activation thresholds, and transcriptional programs central to neuroimmune regulation [60, 61, 62]. Estrogen's immunoregulatory and neuroprotective effects are among the most extensively characterized. Estradiol suppresses NF‐κB signaling, reduces microglial production of pro‐inflammatory cytokines such as TNFα, IL‐1β and IL‐6, and enhances anti‐inflammatory mediators including IL‐10 [63, 64, 65]. In parallel, estrogen promotes neuronal survival by enhancing mitochondrial efficiency, upregulating brain‐derived neurotrophic factor (BDNF), and stabilizing synaptic structure [66, 67, 68].

Testosterone modulates microglial and lymphocyte function through distinct, pathway‐specific mechanisms. Although often broadly characterized as immunosuppressive, androgen signaling selectively shapes inflammatory and regulatory programs rather than uniformly suppressing immunity [69, 70]. In microglia, testosterone signaling via androgen receptors reduces TNFα and IL‐1β production, decreases MHC class II expression, and dampens phagocytic activity [71, 72]. Testosterone also suppresses Th1 and Th17 differentiation while promoting regulatory T cell expansion, leading to reduced IFNγ and IL‐17 secretion [70, 73]. In B cells, androgen signaling limits maturation and antibody production [74]. Clinically, androgen deficiency in males is associated with heightened inflammatory signatures and exaggerated T cell responses, which can be reversed by testosterone replacement therapy [74, 75]. Conversely, lower circulating testosterone levels in females are associated with stronger adaptive immune responses, consistent with the female‐biased prevalence of antibody‐ and T cell–mediated autoimmune diseases [75, 76].

Taken together, current evidence supports the general principle that females tend to mount more vigorous innate and adaptive immune responses, whereas males exhibit stronger immunoregulatory biases in peripheral immunity. However, robust human data defining sex‐biased baseline and inflammatory states within specific CNS‐resident cell types remain limited and, in some cases, in conclusive [77, 78]. Hormone‐dependent immune modulation thus represents a critical axis influencing susceptibility, disease activity, and recovery in neuroinflammatory conditions, but claims regarding sex‐biased baseline neuroinflammation require further mechanistic validation in human CNS tissue.

#### 2.1.2 Sex Chromosomes and Genetic Factors

Beyond hormonal influences, sex chromosome complements (XX, XY and variations) exert cell‐intrinsic effects on immune and neuroimmune function. The X chromosome is particularly enriched for immune‐related genes, including *TLR7*, *TLR8*, *IRAK1*, *BTK*, *CXCR3*, and *FOXP3*. Although one X chromosome undergoes inactivation in females, ~15% of X‐linked genes escape inactivation and remain biallelically expressed [79], immune‐related genes among them. The most studied escapees are *TLR7* and *TLR8*, encoding pattern‐recognition receptors that play central roles in antiviral and inflammatory signaling [80, 81]. Because these genes lack Y‐linked homologs, females effectively carry a higher functional gene dosage of these immune genes, resulting in enhanced basal and ligand‐induced TLR7/8 signaling and stronger type I IFN responses [81, 82].

Elevated TLR7 activity in females promotes plasmacytoid dendritic cell activation, increased IFNα/β production and enhanced B cell maturation and antibody class switching [77, 83, 84, 85, 86]. These mechanisms contribute directly to female‐biased autoimmunity, particularly systemic lupus erythematosus (SLE), where gain‐of‐function variants or duplications of *TLR7* drive severe disease [87]. TLR7‐driven interferon programs are also activated in neuroinflammatory contexts and microglial antiviral responses [88], although CNS‐specific consequences remain under active investigation. Females also carry two copies of immunoregulatory genes such as *FOXP3*, although escape from X‐inactivation appears to be cell‐type dependent [88]. In addition, the X chromosome encodes ~118 microRNAs, compared with only four on the Y chromosome [89]. Many of these microRNAs are immune‐modulatory, regulating cytokine signaling, antigen presentation and T cell activation, thus expanding post‐transcriptional immune regulatory capacity in females.

Despite its limited gene content, the Y chromosome encodes regulators with emerging roles in epigenetic processes relevant to neurobiology and immunity. *UTY* (Ubiquitously Transcribed Tetratricopeptide Repeat–Containing Protein, Y‐Linked; also known as lysine demethylase 6C, KDM6C), the Y‐linked member of the KDM6 family of histone H3 lysine‐27 demethylases, exhibits markedly reduced catalytic activity relative to its X‐linked paralog KDM6A (*UTX*) but is thought to exert important non‐catalytic regulatory functions in chromatin organization and gene expression [90, 91]. KDM5D (*JARID1D/SMCY*) is a functional H3K4me3 demethylase that represses transcription at target promoters [92]. Recent in vivo studies demonstrate that disruption of the *Uty* locus in mouse hematopoietic cells alters chromatin accessibility and gene expression in cardiac monocytes and macrophages, skewing them toward profibrotic phenotypes and exacerbating heart failure, phenocopying effects of mosaic loss of the Y chromosome [93]. While these findings establish that Y‐linked epigenetic regulators can shape myeloid cell states in vivo, their roles in classical inflammatory polarization and CNS immunity remain incompletely defined.

The Y chromosome also influences neuroimmune function indirectly through neural pathways. The sex‐determining gene *SRY* is expressed in catecholaminergic regions of the brain in rodents and humans, where it regulates transcription of enzymes controlling dopamine and noradrenaline synthesis [94, 95, 96]. Because catecholamines modulate both microglial behavior and peripheral immune responses [97, 98], *SRY*‐dependent differences in neurotransmitter production represent a plausible, though as yet untested, mechanistic route linking Y chromosome biology to neuroimmune regulation. Evidence from sex chromosome aneuploidies further supports a role for chromosome dosage in shaping immune risk. Individuals with Klinefelter syndrome (47,XXY) exhibit increased prevalence of several female‐predominant autoimmune diseases, including SLE, rheumatoid arthritis, Sjögren's syndrome, and autoimmune thyroid disease [99]. Notably, males with Klinefelter syndrome display approximately a 14‐fold increased risk of SLE compared with 46,XY males, approaching the risk observed in 46,XX females [100]. In contrast, data on autoimmune risk in Turner syndrome (45,X) are limited, whereas individuals with 47,XXX are overrepresented among patients with SLE and Sjögren's syndrome, consistent with an X chromosome gene‐dosage effect [101]. Functionally, neutrophils and monocytes from 47,XXY individuals produce higher levels of IL‐6, IL‐8, TNFα, and IL‐1β following TLR stimulation than cells from 46,XX females and often 46,XY males, indicating heightened innate inflammatory responsiveness [102].

Together, these data support a model in which X‐linked escape genes, Y‐linked regulators such as UTY, KDM5D, and SRY, and circulating sex hormones jointly shape sex‐biased immune and neuroinflammatory phenotypes. While sex chromosomes introduce intrinsic differences in microglial activation, antiviral signaling, and cytokine production, chromosomal complement alone cannot explain the full diversity of sex‐based phenotypes observed in neurocognitive and neuroinflammatory disorders. Instead, these phenotypes emerge from the integration of chromosomal effects with hormonal, epigenetic, metabolic, and environmental influences.

## 3 Metabolic Orchestrators of Neuroinflammatory Circuitry

A third determinant of neuroimmune divergence has come into focus: metabolism, the biochemical engine that governs how immune cells sense danger and execute inflammatory programs. Mounting evidence indicates that metabolic pathways, ranging from mitochondrial stress responses to lipid and nucleotide remodeling, are not only sexually dimorphic but actively shape male–female differences in neuroinflammatory vulnerability and disease progression. Metabolism does not merely support immune function; it fundamentally influences immune trajectory and persistence. Inflammatory responses impose rapid energetic and biosynthetic demands, requiring coordinated metabolic adaptation to sustain cellular homeostasis.

Pattern‐recognition receptors, including TLRs, TREM2, and the cGAS–STING pathway, converge on NF‐κB and HIF‐1α signaling, thereby directly linking innate immune sensing to metabolic reprogramming [103, 104, 105, 106, 107, 108, 109, 110, 111]. Upon activation, immune cells such as macrophages, dendritic cells, and microglia undergo coordinated metabolic remodeling to support effector functions, cytokine production, and stress responses [112, 113, 114, 115, 116].

Importantly, these immunometabolic programs are strongly shaped by sex hormones and sex chromosome complement, resulting in distinct baseline metabolic and immune states in males and females [117]. Systems‐level analyses in humans consistently show that females exhibit stronger type I interferon (IFN‐I) and antiviral signatures, suggesting an IFN‐linked metabolic bias toward glycolytic and antiviral programs [118]. In contrast, several studies have observed males more frequently display inflammatory and lipid‐associated signatures in human macrophages and mouse models, consistent with greater reliance on lipid utilization and inflammasome activation [119]. These differences are particularly relevant in the brain, which comprises only ~2% of total body mass yet consumes ~20% of resting energy to maintain ion gradients, synaptic transmission, and Na^+^/K^+^‐ATPase activity [120]. Neurons depend on metabolic coupling with astrocytes, oligodendrocytes, and microglia across mammalian systems, creating a tightly integrated energetic ecosystem that intersects directly with local immune regulation [121].

Immune activation in the CNS therefore carries a high metabolic cost. Upon activation, microglia and astrocytes shift toward glycolysis‐driven energy production and mobilize lipid stores to generate inflammatory mediators, remodel membranes, and sustain cytokine synthesis across mammalian systems [122, 123]. In parallel, extracellular metabolites—including purines, lactate, and bioactive lipids—fluctuate and feed back into microglial signaling pathways through receptors such as P2X7, adenosine receptors, and TREM2 in preclinical models, with emerging relevance in human disease [124, 125, 126, 127, 128, 129]. Even modest metabolic perturbations can thus have outsized neuroimmune consequences. Disrupted purine metabolism, for example, can amplify cytokine release and drive chronic neuroinflammation in preclinical models of AD and MS, with converging evidence from human disease studies [130, 131, 132].

Against this backdrop, biological sex emerges as a critical modifier of neuroimmune metabolism. Sex hormones and chromosomal complement shape both baseline metabolic programs and immunometabolic responses to stress, injury, and infection in the brain. These differences likely contribute to the well‐established sex biases observed across neuroinflammatory disorders in humans, including MS, AD, and autoimmune encephalitis [117, 133]. In the sections below, we examine how sex influences three central axes of neuroimmune metabolism—glycolytic reprogramming, lipid sensing, and purine metabolism—each of which differentially tunes inflammatory versus reparative programs in the CNS.

### 3.1 Glycolytic Reprogramming

Innate immune activation is tightly coupled to metabolic reprogramming. In response to pathogens, damaged neurons, or misfolded proteins, innate immune cells, including macrophages, dendritic cells, and microglia, undergo coordinated metabolic shifts to meet the energetic and biosynthetic demands of inflammation. Engagement of PRRs such as TLRs, TREM2, and the cGAS–STING pathway activates NF‐κB and HIF‐1α signaling, leading to upregulation of glucose transporters and glycolytic enzymes across experimental systems [107, 134, 135, 136]. This promotes a transition from oxidative phosphorylation (OXPHOS) to aerobic glycolysis, a Warburg‐like state that enables rapid ATP production and provides intermediates required for cytokine synthesis, redox balance, and membrane remodeling across immune cells and experimental systems [134, 137]. Glycolytic reprogramming is now recognized as a hallmark of inflammatory microglia and has been observed in both mouse models and human studies of neurodegenerative disease, including PD [138, 139, 140], AD [141, 142, 143], and ALS [144, 145]. Notably, early glycolytic engagement precedes overt inflammatory commitment in mouse microglia, occurring before cytokine amplification and morphological transformation [146, 147, 148].

Metabolic intermediates generated during glycolysis also function as signaling nodes that shape inflammatory output. Succinate stabilizes HIF‐1α and promotes IL‐1β production [149, 150]; lactate modulates immune tolerance and chronic inflammation [151, 152]; and itaconate, derived from the TCA cycle intermediate citrate, acts as a counter‐regulatory brake by suppressing IFN‐I and NF‐κB signaling in mouse and human immune cells [153, 154]. Through these metabolites, glycolysis both fuels and fine‐tunes innate immune responses, influencing the amplitude and duration of CNS inflammation.

Sex differences further modulate glycolytic programming and its immunological consequences. Multi‐omics studies demonstrate that female human immune cells exhibit higher basal IFN‐I and antiviral signatures, consistent with a glycolytic‐leaning metabolic bias [62, 118, 155, 156, 157]. In contrast, male myeloid cells preferentially engage lipid‐catabolic and inflammasome‐associated pathways in both mouse models and human studies, reflecting a tendency toward oxidative or lipid‐driven metabolism [119, 158, 159, 160]. In the CNS, these systemic tendencies may translate into sex‐biased microglial activation thresholds in experimental models, as well as emerging support from human studies, with females more prone to IFN‐linked glycolytic reprogramming and males favoring NLRP3‐dependent, lipid‐coupled inflammation [161, 162, 163, 164]. Together, these findings position glycolytic metabolism as a central nexus linking sex, immunity, and neuroinflammatory susceptibility.

### 3.2 Lipid Sensing and Metabolic Inflammation

Innate immune cells in the CNS, particularly microglia, are highly sensitive to changes in the lipid milieu. This sensitivity is mediated by a broad repertoire of lipid‐sensing receptors, including TLRs, CD36, TREM2, prostaglandin receptors, phospholipid receptors, and endocannabinoid receptors in mouse models, with corroborating evidence from human studies [165, 166, 167]. Through these receptors, microglia detect lipids derived from pathogens, damaged neurons, and myelin debris. In neurodegenerative disease models such as AD, lipid‐associated signals, including cholesterol crystals, oxidized phospholipids, and amyloid–lipid complexes, drive inflammatory activation by promoting inflammasome assembly, NF‐κB–dependent cytokine production, and impaired phagocytic clearance [168, 169, 170]. Beyond acting as inflammatory triggers, lipid metabolites actively instruct immune trajectories across murine and human immune systems. Oxidized LDL and cholesterol crystals promote inflammasome activation [171, 172]; eicosanoids such as prostaglandins and leukotrienes shape cytokine production and leukocyte recruitment [173]; and specialized pro‐resolving mediators (SPMs), including resolvins and maresins, actively terminate inflammation and promote tissue repair [174]. In mouse microglia, defective lipid handling, as exemplified by impaired TREM2 signaling, induces metabolic stress, defective phagocytosis, and chronic pro‐inflammatory states that exacerbate neurodegeneration [170, 171, 172]. Collectively, lipid sensing integrates environmental danger cues with metabolic state, positioning lipid pathways as central regulators of microglial inflammatory function.

Sex differences further shape how microglia interpret and respond to lipid cues. Sex hormones and chromosomal complement modulate the activity of multiple lipid‐sensing receptors, including TLRs, CD36, TREM2, and components of the endocannabinoid system, resulting in sex‐biased inflammatory tendencies across murine and human immune systems [175, 176, 177]. In human AD, interactions between APOE‐ε4 and TREM2 produce stronger transcriptional and pathological consequences in females [178, 179], suggesting differential tuning of key lipid‐sensing checkpoints. Lipid mediator biosynthesis is also sexually dimorphic in humans: androgens enhance leukotriene pathways and modulate prostaglandin production [180, 181], implicating eicosanoid balance itself as a contributor to sex‐specific inflammatory trajectories. Together, these findings indicate that sex shapes both lipid sensing and downstream lipid‐driven immunometabolic output, influencing vulnerability to neuroinflammation and neurodegeneration.

### 3.3 Purine Metabolism and the ADA Axis

Alongside lipid‐mediated pathways, purine metabolism represents a second major sex‐biased axis through which microglia integrate metabolic stress with innate immune activation. denosine deaminases catalyze the deamination of adenosine and deoxyadenosine to inosine and deoxyinosine, thereby shaping intracellular and extracellular purine pools. In humans, ADA1 is broadly expressed and primarily intracellular, whereas ADA2 is secreted by myeloid cells and functions predominantly in the extracellular space [28, 182, 183]. In the CNS, microglia and perivascular macrophages are repeatedly exposed to purine‐rich microenvironments generated by neuronal injury, demyelination, and metabolic stress in human inflammatory disease contexts, positioning ADA activity as a key determinant of microglial inflammatory set point [184]. Microglia express a diverse repertoire of purinergic receptors, including P2X7, P2Y receptors, and adenosine receptors (A1–A3), that enable detection of nucleotides and nucleosides released during tissue damage across murine and human immune systems [185, 186, 187]. ATP–P2X7 signaling promotes NLRP3 inflammasome activation and IL‐1β release, whereas adenosine receptor signaling can suppress or sustain inflammation depending on receptor subtype and ligand concentration, defined in murine immune cells and conserved in humans [188, 189, 190]. ADA1 and ADA2 sculpt this extracellular landscape: ADA1 maintains intracellular purine homeostasis, while ADA2 clears extracellular adenosine and deoxyadenosine [182, 183]. Reduced ADA2 activity biases signaling toward persistent purine exposure, favoring inflammasome activation, heightened IFN‐I output, and chronic neuroinflammation [28, 31].

In human cells, loss of ADA2 profoundly rewires purine metabolism and engages antiviral‐like innate immune programs [28]. Accumulated extracellular deoxyadenosine is alternatively deaminated by ADA1, increasing deoxyinosine levels, which competitively inhibit methionine adenosyltransferase (MAT) and reduce S‐adenosylmethionine (SAM) [28]. SAM depletion drives global hypomethylation and derepression of human endogenous retroviruses (HERVs), whose transcripts and nucleic‐acid intermediates are sensed by RIG‐I/MDA5 and the cGAS–STING pathway, culminating in robust IFNβ induction [28]. In the CNS, repeated fluctuations in ATP and nucleoside levels during injury and degeneration may predispose microglia with impaired ADA2 activity to HERV derepression and IFN‐I activation in murine models [185, 191]. Consistent with this model, HERV transcripts and proteins are elevated in human AD and MS [192, 193, 194], HERV‐W envelope protein contributes to inflammation in human MS [195], and IFN‐I–responsive microglial states, defined in experimental models and supported by human studies, are increasingly implicated in synapse loss and neurodegeneration in AD and ALS [196, 197].

Emerging evidence further indicates that purine metabolism and ADA activity are sexually dimorphic. Systems‐immunology analyses show that females more frequently exhibit lower ADA/ADA2 activity accompanied by higher basal HERV expression, elevated interferon‐stimulated gene (ISG) signatures, and enhanced IFN‐I programs in both healthy individuals and patients with COVID‐19 [194]. These findings align with the ADA2–HERV–IFNβ circuit described above and suggest that modest reductions in ADA activity may preferentially prime antiviral‐like programs in females. More broadly in humans, females display greater sensitivity to systemic metabolic stress—including glycemic, vascular, and hepatic perturbations—with distinct sex‐specific metabolite signatures across metabolic states [159, 198]. Reviews of sex‐biased immunometabolism similarly indicate that female immune systems more readily couple metabolic fluctuations, such as altered lipid or nucleotide flux, to stronger inflammatory outputs [78, 199].

These sex‐biased immunometabolic programs parallel disease‐specific patterns across neurodegenerative disorders. In AD, which disproportionately affects females, female microglia in both APP/PS1 mice and human tissue exhibit a pronounced shift toward aerobic glycolysis (elevated ECAR, PFKFB3 expression, and lactate production), accumulation of pro‐inflammatory succinate, impaired phagocytosis, and increased amyloid burden [160]. By contrast, male microglia more frequently maintain oxidative metabolism and phagocytic function [160, 200]. In human MS, more prevalent in females, glycolytic and IFN‐skewed immune programs may underlie heightened autoimmunity, whereas males more often develop chronic‐active lesions with iron‐laden microglia consistent with sustained inflammasome activation [201, 202]. Conversely, human PD shows male predominance, where estrogen's neuroprotective and mitochondria‐stabilizing effects may confer female resilience, while male‐biased lipid and inflammasome pathways amplify disease risk [19, 203, 204]. In ALS, recent multi‐omic profiling reveals greater disruption of immune, extracellular matrix, and mitochondrial pathways in male patients compared with females [205]. Together, these findings support a model in which sex‐specific metabolic programs—from PFKFB3‐driven glycolysis in females to androgen‐associated lipid signaling in males—shape divergent neuroinflammatory trajectories across AD, PD, MS, and ALS.

## 4 Metabolic Control of Innate Immune Signaling in Neuroinflammation

Metabolism and innate immune signaling have traditionally been framed as parallel but independent regulatory layers. Recent studies support an integrated model in which metabolic enzymes, nucleotide flux, and post‐translational signaling checkpoints directly shape inflammatory identity across human immune cells. These discoveries—spanning nucleotide‐controlled T‐cell cytokine fate [183], a kinase‐dependent licensing switch for cyclic cGAS–STING activation [203], and a purine‐driven endogenous retrovirus–type I interferon circuit with sex‐biased features [28, 32], collectively position metabolism as a first‐order determinant of immune response amplitude and persistence. Importantly, these mechanisms intersect with central neuroimmune pathways, including meningeal and brain‐border T‐cell activity, microglial DNA sensing, nucleoside‐reactive interferon programs, and sex‐biased inflammatory set points that influence neurodegenerative risk. In this section, we synthesize these mechanistic nodes and outline how they form an integrated, and potentially targetable, framework for understanding maladaptive CNS inflammation.

### 4.1 ISOC1 Couples Nucleotide Metabolism to Inflammatory Fate in Human Memory T Cells

Kushnareva et al. [204] investigated how gene programs shape effector function in human Th1‐like memory CD4^+^ T cells, a subset enriched in IFN‐experienced individuals. Using CRISPR/RNAi perturbations integrated with transcriptomics and targeted metabolomics, the study identified ISOC1 (Isochorismatase Domain–Containing Protein 1) as a previously underappreciated metabolic gatekeeper. ISOC1 knockdown blunted IFNγ and IL‐17 production, and metabolomic profiling revealed broad defects in pyrimidine and purine metabolism, including depleted nucleotide pools and altered redox balance. Mechanistically, ISOC1 deficiency constrained glycolytic and mitochondrial support for nucleotide biosynthesis, functionally limiting cytokine output upon recall stimulation. These findings position ISOC1 at the intersection of nucleotide anabolism and pro‐inflammatory effector programming, emphasizing that immune fate depends not only on canonical transcription factors but also on metabolic enzymes that license nucleotide availability. Beyond defining a single gene, this work established a generalizable pipeline integrating functional genomics and metabolomics to connect metabolic control points to human T‐cell function.

Insights from ISOC1‐dependent metabolic control in memory T cells offer a lens through which to interpret how nucleotide‐stress pathways might shape neuroinflammatory circuitry. Increasing evidence supports the relevance of metabolic gatekeepers identified in peripheral immune cells for understanding CNS inflammation [206]. In particular, ISOC1‐dependent control of IFNγ and IL‐17 production has implications for meningeal and blood–brain barrier (BBB) compartments that influence microglial state and synaptic integrity. Conditions that impose nucleotide stress at CNS interfaces, including aging, vascular dysfunction, chronic infection, hypoxia, mitochondrial injury, or BBB leakage, could plausibly skew T‐cell cytokine programs. Heightened IFNγ and IL‐17 in these settings may reinforce microglial transitions into disease‐associated or interferon‐responsive states described in neuroinflammatory disease and aging, thereby potentiating microgliosis and aberrant synaptic remodeling.

More broadly, the central principle emerging from this study, that nucleotide metabolism licenses inflammatory fate, aligns with core features of microglial biology. Microglia integrate extracellular ATP and adenosine to regulate chemotaxis, phagocytosis, and inflammasome activation [207]. Thus, limited nucleotide availability, diminished biosynthetic capacity, and redox stress may act as conserved metabolic signals that shape inflammatory behavior across immune cell lineages.

Disease contexts further illustrate translational relevance. In the APP/PS1 mouse model of AD, IFNγ–driven immune signaling promotes microglial activation during early stages of pathology, and Th1‐like cytokines exacerbate synaptic dysfunction [208], features that could be intensified by ISOC1‐tuned cytokine output at the BBB. In MS patients, IL‐17 and IFNγ are key drivers of lesion pathogenesis [209], raising the possibility that nucleotide‐regulated effector programs contribute to inflammatory fluctuations during relapse. In PD and ALS, pervasive mitochondrial dysfunction and metabolic stress [210] may similarly constrain nucleotide availability, potentially biasing both T‐cell and glial responses toward chronic, low‐grade inflammation.

The methodological blueprint established in the ISOC1 study, integrating CRISPR perturbations with metabolomics, is readily applicable to human iPSC‐derived microglia to test whether ISOC1 or adjacent metabolic enzymes tune IFN‐I programs, phagocytic capacity, synapse engulfment, and interactions with purinergic and cGAS–STING pathways. From a translational perspective, ISOC1 highlights nucleotide metabolism as a potentially modifiable regulator of neuroinflammation that could be targeted without broad immunosuppression. In neurodegenerative cohorts, boosting nucleotide synthesis through salvage‐pathway precursors or one‐carbon donors could, in principle, normalize pathologic IFNγ/IL‐17 output at CNS borders and reset microglial states. Conversely, in cytokine‐rich environments such as active MS lesions, fine‐tuning nucleotide availability or reactions proximal to ISOC1 may constrain excessive Th1/Th17 effector output while preserving antiviral defenses. Nucleotide–redox signatures in CSF or blood may provide practical pharmacodynamic biomarkers of pathway engagement [211]. In combination with purinergic interventions (e.g., P2X7 or A2A modulation) linked to microglial reactivity, ISOC1‐informed strategies could enable multi‐node regimens that target both intracellular nucleotide constraints and extracellular nucleotide signaling.

Direct evidence for sex‐based differences in ISOC1 expression is currently limited, but several lines of evidence suggest potential modulation. Rodent studies link ISOC1 to testis (and eye) development [212, 213], consistent with possible hormonal regulation. More broadly, sex hormones and sex chromosomes shape immune gene networks, and disease risk and inflammatory programs diverge in males and females. For example, IFNγ variants affect MS risk in patients in a sex‐dependent manner [214], whereas in AD, which is more prevalent in females, female microglia in APP/PS1 mice adopt a glycolytic, pro‐inflammatory phenotype [160]. If ISOC1 limits nucleotide supply for cytokine synthesis, these contexts could plausibly modulate inflammation in a sex‐dependent manner. Accordingly, ISOC1‐targeted or nucleotide‐boosting strategies may show sex‐differential efficacy, motivating direct testing in male versus female immune cells and models.

### 4.2 DAPK3 As a cGAS–STING Licensing Kinase: Mechanistic Insights and Neuroimmune Implications

Death‐Associated Protein Kinase 3 (DAPK3) has been identified as an essential upstream kinase for cGAS–STING activation that supports antitumor innate immunity [209]. Using a 1001‐gene loss‐of‐function screen in human umbilical vein endothelial cells (HUVEC), DAPK3 emerged as a positive regulator of the cGAS–STING axis. Mechanistically, DAPK3 maintains basal STING stability by inhibiting K48‐linked polyubiquitination, thereby limiting proteasomal degradation. Upon DNA sensing (e.g., 2′,3′‐cGAMP stimulation), DAPK3 promotes STING K63‐linked polyubiquitination and assembly of the STING–TBK1 signalosome. Phosphoproteomics identified LMO7 as a DAPK3 substrate; phosphorylation of LMO7 was required for LMO7–STING interaction and the K63‐linked ubiquitin editing that licenses downstream IFNβ transcription. In murine syngeneic tumor models, DAPK3 loss suppressed interferon‐stimulated gene programs, impaired recruitment of CD103^+^ dendritic cells and cytotoxic T cells, accelerated tumor growth, and blunted responses to chemo‐immunotherapy. Collectively, these data position DAPK3 as a dual‐mode STING rheostat: preserving STING at baseline while catalyzing activating ubiquitin‐state transitions during innate sensing.

Increasing evidence implicates cGAS–STING signaling in CNS aging and neurodegeneration, including AD [215]. In aging and AD human samples and mouse models, STING activation in microglia has been linked to reactive transcriptional states, synaptic loss, neurodegeneration, and cognitive decline [216], whereas genetic or pharmacologic disruption of cGAS/STING in AppNL‐G‐F/hTau double‐knock‐in mice ameliorates pathology and improves behavior [217]. Human AD tissue has been reported to show elevated STING immunoreactivity in proximity to plaques and microvasculature, consistent with sterile DNA stress (e.g., mitochondrial DNA leakage, micronuclei, or endogenous retroelements) fueling innate activation [215]. Within this framework, the DAPK3 study adds a mechanistic lever: a kinase‐dependent licensing step that controls STING stability and activation through post‐translational ubiquitin editing (K48 versus K63) via LMO7.

In the brain, where chronic, low‐grade DNA danger signals may persist, a DAPK3‐dependent licensing step could set the threshold for microglial STING activation. Higher DAPK3 activity could facilitate STING–TBK1 assembly and sustain IFN‐I programs, whereas reduced DAPK3 activity could destabilize STING and attenuate signaling. This bidirectional control aligns with evidence that tonic IFN‐I signaling in murine microglia worsens AD‐like phenotypes and that IFNAR blockade can rescue synaptic and cognitive measures in models [218]. Beyond AD, both ALS patient‐derived cells and a mouse model of C9orf72 repeat‐associated ALS also feature DNA damage and mitochondrial stress, conditions favorable for cGAS–STING engagement [219], suggesting that a DAPK3‐tuned STING checkpoint could generalize across neurodegenerative contexts. In parallel, work implicating purine metabolism as an upstream driver of IFN‐I (the ADA2 axis) in humans positions DAPK3 as a convergent checkpoint on the STING arm of the same interferon‐centered circuitry [216].

These observations motivate the hypothesis that DAPK3 and its substrate circuit (LMO7‐dependent ubiquitin editing) could represent therapeutic entry points in contexts where cGAS–STING–IFN‐I signaling is pathogenic. If excessive STING activity contributes to AD/ALS/aging phenotypes, DAPK3 inhibition could, at least in principle, reduce K63‐linked licensing and dampen IFN‐I programs, potentially offering finer control than direct STING antagonism. Conversely, augmenting DAPK3‐mediated stabilization might be relevant in settings requiring enhanced antiviral responses, though such approaches are less likely to be desirable in chronic neurodegeneration. The defined phosphorylation site on LMO7 and the K48‐to‐K63 ubiquitin switch suggest tractable intervention points (protein–protein interactions or E3‐ligase pathways) that could modulate STING without globally suppressing innate immunity. Potential pharmacodynamic markers include phospho‐LMO7, the ratio of K63‐ubiquitylated to total STING, and DAPK3 kinase activity in peripheral myeloid cells or iPSC‐derived microglia.

Sex‐stratified analyses of DAPK3 in human neurodegenerative disease remain limited; however, several lines of evidence suggest that DAPK3 signaling is hormonally and transcriptionally modulated in a sex‐dependent manner in rodent and human sytems [220, 221, 222]. DAPK3 has been described as androgen responsive and can enhance androgen receptor–mediated transcription as a co‐activator within AR signaling complexes [222]. Consistent with androgenic regulation, *DAPK3* expression was higher in males in a transcriptomic survey of mitochondrial and apoptotic genes in the hearts of F344 rats, clustering within a male‐biased stress response program [223]. Although peripheral, these data provide precedent for sex‐linked regulation of DAPK3 expression and motivate investigation in CNS‐resident immune compartments.

Integrating sex‐biased epidemiology and immune programs with DAPK3–STING biology suggests testable predictions. For example, if DAPK3 expression or activity differs by sex in CNS myeloid cells, DAPK3 inhibition could yield sex‐differential effects on STING‐dependent IFN‐I outputs, potentially producing steeper IFN‐I normalization in IFN‐high contexts. Such hypotheses require direct testing in human brain tissue and in sex‐stratified iPSC‐microglia models. Biomarker strategies for DAPK3–STING modulation should therefore be sex stratified, incorporating phospho‐LMO7, STING ubiquitin‐state measures, and interferon signatures as pharmacodynamic readouts.

Finally, accumulating evidence indicates that the pathological consequences of STING signaling in the CNS are shaped by cellular context, mitochondrial stress, and interferon sensitivity, not kinase control alone [215]. Several of these contextual determinants differ by biological sex. AD exhibits female‐biased interferon‐responsive microglial states, whereas PD and ALS show greater male vulnerability to mitochondrial and oxidative stress [215, 224, 225, 226, 227]. Notably, APOE ε4 and female sex have been reported to amplify tau‐induced microglial cGAS–STING–IFN‐I responses in APOE4‐R47H human P301S mutant tau mice, providing direct evidence that STING pathway output is sex modulated in neurodegeneration [228]. In this framework, DAPK3‐dependent stabilization and licensing of STING may represent a necessary but insufficient step for IFN‐I induction, with downstream amplitude further tuned by sex‐biased immunometabolic programs.

### 4.3 The ADA Axis Links Purine Nucleosides to IFN‐I and Sex Bias

A novel purine‐to‐interferon pathway was recently described in which extracellular deoxyadenosine and deoxyinosine accumulate when ADA2 activity is reduced in HUVEC [28]. These nucleosides are taken up and metabolized in a manner that inhibits methionine adenosyltransferase, lowering S‐adenosylmethionine (SAM), reducing DNA methylation, and derepressing human endogenous retroviruses (HERVs). HERV‐derived transcripts engage RNA‐sensing pathways (RIG‐I/MDA5–MAVS), inducing IFNβ and interferon‐stimulated genes. Genetic or pharmacologic reduction of ADA2 activity, as well as nucleoside supplementation, was sufficient to trigger IFN‐I programs, whereas restoring ADA activity or disrupting nucleic‐acid sensing curtailed responses [28]. This work established ADA2 as a metabolic brake on an innate antiviral circuit rooted in purine catabolism, epigenetic regulation, and HERV sensing.

Subsequent human cohort analyses extended this framework to sex differences [227]. Across cohorts, females exhibited lower ADA activity and higher HERV/ISG signatures, consistent with stronger basal IFN‐I priming. Together, these studies position ADA1/ADA2 as upstream tuners of tissue interferon set points and provide a biochemical rationale for sex‐biased innate immune activation across tissues, with direct relevance to neuroinflammation.

CNS‐resident glia are highly responsive to purine cues [185]. During neuronal stress, extracellular ATP activates P2X7 and promotes NLRP3 inflammasome signaling, whereas ectonucleotidases CD39 and CD73 regulate adenosine levels and, in turn, ADA1 and ADA2 metabolize nucleosides [229, 230]. These findings suggest that when ADA2 is insufficient, extracellular deoxyadenosine/deoxyinosine accumulation may prime IFN‐I via HERV derepression, potentially shifting microglia toward interferon‐responsive states observed in aging and AD. In MS, tonic IFN‐I can be beneficial or harmful depending on context; ADA–nucleoside signaling at CNS borders could contribute to this bidirectionality by modulating baseline interferon tone. In PD and ALS, mitochondrial stress and nucleic acid instability could synergize with ADA‐low states to sustain aberrant IFN programs that impair neuronal survival and microglial function. Critically, the observed sex bias that is, lower ADA activity and higher IFN priming in females, offers a plausible mechanistic contributor to female‐skewed AD and MS risk and to sex‐differential therapeutic responses.

From a translational perspective, three complementary avenues emerge. First, restoring the ADA2 brake on nucleoside accumulation could, in principle, lower deoxyadenosine/deoxyinosine levels and attenuate IFN‐I signatures. Candidate approaches include recombinant ADA2, gene therapy strategies informed by DADA2, or small‐molecule ADA‐enhancing strategies, initially tested peripherally with evaluation of central effects through interferon‐state profiling and clinical outcomes. Given consistently lower ADA activity in females, sex‐aware dosing and stratification would likely be required. Second, if direct ADA restoration proves impractical, intervention along the HERV–sensor axis remains an alternative, including modulation of RNA‐sensing pathways (RIG‐I/MDA5), downstream IFNAR/JAK–STAT signaling, or HERV biogenesis, ideally with CNS‐tuned delivery to avoid broad immunosuppression. These strategies could be complemented by purinergic modulators (P2X7 or A2A) that shape the ATP/adenosine signals co‐regulating microglial activation. Third, stratification frameworks incorporating ADA activity, deoxyadenosine/deoxyinosine levels, HERV transcripts, and ISG signatures, contextualized by sex and hormonal status, may guide eligibility and pharmacodynamic monitoring in early‐phase efforts targeting ADA restoration or HERV sensing pathways, with the expectation that ADA‐low/IFN‐high states (more common in females) may represent particularly responsive subgroups.

## 5 Immunometabolic Drivers of Neuroinflammation

The studies synthesized above highlight a recurrent theme: innate immune activation in neuroinflammatory disease is strongly shaped by underlying metabolic programs and is modulated in sex‐specific ways. Collectively, these findings underscore the value of examining upstream metabolic cues that bias immune cell fate and inflammatory trajectories. In keeping with this broader immunometabolic landscape, we next focus on bioactive lipids, particularly lysophosphatidylcholine (LPC) species, as regulators of neutrophil behavior and systemic inflammation. Recent work from our laboratory indicates that LPC dysregulation emerges in human post‐viral inflammatory syndromes, including neurocognitive long COVID (unpublished), and during autoimmune‐like immune‐related adverse events (irAEs) following immune checkpoint blockade (ICB) therapy [231]. In these settings, declines in LPC 18:2 correlate with heightened neutrophil activation and persistent inflammatory tone. Preliminary data in AD models further suggest that circulating LPC 18:2 declines in aged AD mice (unpublished) coincident with rising peripheral neutrophil counts, consistent with a lipid–immune imbalance that could amplify early neuroinflammation [232]. In addition, intraperitoneal LPC administration in 5xFAD and 3xTg‐AD mice has been reported to reduce Aβ plaque burden and attenuate gliosis in 5xFAD mice [233]. Together, these observations across distinct disease contexts support the idea that LPC species may function as broader immunometabolic indicators of inflammatory load, with emerging evidence linking circulating LPC levels to peripheral immune activation and early features of AD in human cohorts [234, 235]. Although direct causal links between circulating LPC changes and microglial state transitions remain to be established, these associations raise the possibility that lipid–immune signatures intersect with early microglial remodeling.

LPCs are single–acyl‐chain phospholipids generated by partial hydrolysis of phosphatidylcholine, typically via phospholipase A_2_ activity [236]. LPC 18:2 contains a linoleic acid (18:2) chain and is a prominent circulating LPC species in both humans and mice [237, 238, 239, 240, 241]. LPCs are enriched in oxidized lipoproteins and inflamed tissues, where they act as signaling molecules and exert context‐dependent immunomodulatory functions [236, 242, 243]. LPCs can function as chemoattractants for murine T cells, human monocytes, and human neutrophils [244] and can enhance endothelial expression of adhesion molecules such as ICAM‐1 and VCAM‐1 in human cells, potentially facilitating leukocyte recruitment [244, 245]. In monocyte‐derived dendritic cells, LPCs promote maturation, upregulate CD83/CD86, and enhance antigen presentation, functioning as endogenous adjuvant‐like signals that support CD4^+^ and CD8^+^ T‐cell priming [246, 247] LPCs can also activate NLRP3 inflammasomes in human macrophages in certain contexts [248], yet in other settings can promote pro‐resolving macrophage programs as demonstrated in an in vivo mouse model system [249]. Thus, LPC species function as lipid cues that can either amplify or restrain inflammation depending on tissue context, acyl composition, and immune activation state.

### 5.1 LPC 18:2 as a Systemic Regulator of Neutrophil Persistence During ICB‐irAEs

LPC 18:2 has been identified as an endogenous suppressor of severe immune‐related adverse events (irAEs) in patients receiving immune checkpoint blockade therapy (ICB) [231]. irAEs are inflammatory toxicities that arise from loss of self‐tolerance when checkpoint inhibition reinvigorates antitumor T cells, enabling collateral immune‐mediated damage to healthy tissues, often in the setting of inflammatory cytokines such as IL‐6 and TNFα [250]. irAEs can affect nearly any organ system; common manifestations include dermatitis/pruritus, diarrhea/colitis, and endocrinopathies [251]. Across trials and real‐world cohorts, a majority of patients experience any‐grade irAEs, while a substantial subset develop severe grade III/IV toxicities [252]. Although fatal irAEs are uncommon overall, specific syndromes such as ICB‐associated myocarditis can carry high mortality [253].

Longitudinal lipidomic analyses across multiple ICB‐treated patient cohorts revealed that individuals who later developed grade III/IV irAEs exhibited a marked post‐treatment decline in plasma LPC 18:2 [231]. Across both ICB cohorts and orthogonal control datasets, lower LPC 18:2 correlated strongly with higher neutrophil counts, and in irAE cases LPC 18:2 inversely tracked with irAE‐associated neutrophilia [231]. Mechanistically, IL‐6 suppressed LPC 18:2 biosynthesis during inflammatory activation [231]. Importantly, in mouse models of CTLA‐4 blockade and colitis, LPC 18:2 supplementation reduced neutrophil expansion and colonic inflammation without impairing tumor rejection [231].

Ongoing work further suggests a cellular mechanism by which LPC 18:2 may restrain tissue‐damaging inflammation. In preliminary studies, LPC 18:2 promoted caspase‐3/7–dependent human neutrophil apoptosis with minimal induction of NETosis, consistent with an immunologically “silent” resolution program (unpublished) [254]. This provides a plausible mechanistic rationale for the observed reduction in neutrophil‐driven colonic inflammation in ICB‐induced colitis models and supports the broader view that LPC 18:2 can act as a metabolic brake on neutrophil persistence. Collectively, these findings position LPC 18:2 as a context‐dependent regulator of innate inflammatory load during immune checkpoint therapy.

Beyond cancer immunotherapy, converging evidence from the broader LPC literature suggests that LPC biology represents a unifying metabolic–immune axis relevant to chronic inflammation, post‐viral syndromes, and neurodegenerative disease. Circulating LPCs decline across diverse inflammatory states in humans, including sepsis, cardiovascular disease, inflammatory bowel disease, atherosclerosis, and acute SARS‐CoV‐2 infection [239, 240, 255, 256, 257, 258, 259], and lower systemic LPC levels frequently associate with heightened innate immune activation and adverse outcomes. Both long COVID and AD are characterized by persistent innate immune activation, altered systemic lipid metabolism, endothelial dysfunction, and impaired resolution programs [260, 261, 262, 263, 264], contexts in which dysregulated LPC‐mediated pathways could plausibly amplify neurocognitive symptoms. In long COVID, sustained neutrophil activity, mitochondrial stress, and endothelial injury provide a biological setting where LPC‐driven apoptotic programming may be particularly relevant [265, 266, 267]. In AD, early declines in circulating LPCs have been reported alongside exaggerated neuroinflammatory signatures [235, 268], raising the possibility that LPC deficiency may lower the threshold for both peripheral and CNS immune activation.

Finally, LPC biology intersects directly with CNS lipid homeostasis. Docosahexaenoic acid (DHA) and eicosapentaenoic acid (EPA) are long‐chain omega‐3 fatty acids with well‐established cardioprotective and neuroprotective functions [239, 269, 270, 271]. Notably, LPC is a principal esterified carrier that transports DHA and EPA across the blood–brain barrier via MFSD2A [239, 272, 273, 274]. Multiple studies show that, in mice, LPC is required to enrich DHA/EPA in the brain, whereas supplementation with unesterified (free) DHA/EPA does not reliably increase brain levels [273, 274]. Consistent with this, LPC‐mediated DHA enrichment in mice improves spatial learning and memory in experimental systems, whereas free DHA has limited effect in the absence of LPC transport [274]. Because DHA and EPA are largely diet derived and not synthesized in the brain at meaningful levels in humans [275], LPC availability may be a key determinant of intracerebral omega‐3 pools. Supporting this model, mutations in MFSD2A/Mfsd2a impair brain uptake of LPC‐bound lipids and cause neurodevelopmental and cognitive defects in humans and mice [276, 277, 278, 279]. Together, these findings position LPC as a lipid mediator linking peripheral lipid metabolism to CNS resilience and neuroinflammatory set points [275, 280].

### 5.2 A Lipid–Immune Disconnect in Neurocognitive Long COVID

Following acute SARS‐CoV‐2 infection, a substantial fraction of survivors experience at least one unresolved symptom lasting ≥ 3 months, meeting criteria for long COVID [281, 282]. Long COVID is highly heterogeneous and can involve more than 200 symptoms spanning multiple organ systems [283]. The biological mechanisms remain incompletely understood, and no FDA‐approved therapeutics exist. Given this heterogeneity, focusing on individuals with a defined symptom cluster may help clarify mechanistic drivers.

To address this, a longitudinal study of healthcare workers in Los Angeles was initiated during the first wave of the COVID‐19 pandemic. From this cohort, 135 age‐ and sex‐matched participants were selected and grouped into: (i) never infected, (ii) COVID‐19 recovered, or (iii) experiencing one or more neurocognitive symptoms of long COVID. Serological analysis showed that both the neurocognitive long COVID and COVID‐recovered patients showed higher circulating spike IgG than never‐infected controls, consistent with prior exposure to SAR‐CoV‐2 (unpublished data). Notably, the male neurocognitive long COVID participants exhibited significantly higher spike IgG titers compared to recovered individuals, suggesting sex divergence in post‐infection immune regulation in settings of long COVID disease.

Untargeted lipidomics revealed a distinct lipid signature in neurocognitive long COVID participants compared to COVID‐recovered participants, even after multivariate modeling accounting for age and sex. Relative to both never‐infected and COVID‐recovered groups, neurocognitive long COVID was characterized by lower circulating levels of inflammation‐resolving lipid mediators, including specialized pro‐resolving mediators (SPMs) such as maresin 1 (MaR1) and 5‐HEPE (EPA‐derived). These SPMs are central to active resolution programs, including in neuroinflammatory contexts [284, 285]. Neurocognitive long COVID participants also showed reduced circulating LPC 18:2 relative to both comparator groups.

Together, these data suggest that neurocognitive long COVID may involve impaired lipid‐mediated resolution capacity rather than broadly elevated cytokinemia. Consistent with this, we did not observe uniform increases in canonical pro‐inflammatory cytokines in our neurocognitive long COVID subgroup. Differences across long COVID studies likely reflect cohort heterogeneity and sampling context [283, 286, 287]. Our cohort was drawn from the first pandemic wave (vaccine‐naïve, infection‐naïve), and participants were selected for neurocognitive symptoms, factors that may shape immune and cytokine profiles compared with cohorts enriched for recurrent infection or vaccination. These findings support the idea that cytokine measures alone may not reliably distinguish neurocognitive long COVID from recovery and instead highlight lipid mediators—including LPC 18:2 and SPMs—as candidate mechanistic drivers and therapeutic targets in this symptom‐defined subgroup [283, 288]. As many of these lipids are influenced by diet and systemic metabolism, a testable implication is whether targeted nutritional or pharmacologic strategies that restore resolution mediators can improve neurocognitive outcomes.

### 5.3 Metabolites, Microglia, and Sex: Early Divergence Points in Alzheimer's Disease

LPC 18:2 has gained attention not only for immunoregulatory properties but also for inverse associations with cognitive decline and dementia risk in humans [239, 289, 290]. Epidemiological studies report negative associations between LPC 18:2 and subclinical atherosclerosis [290], a vascular phenotype linked to dementia risk [291], positioning LPC 18:2 as a candidate metabolic indicator of neurocognitive vulnerability. Complementing these findings, APOE‐ε4 carriers exhibit broad disruptions in glycerophospholipid and ether‐lipid metabolism that track with amyloid burden, tau pathology, and cognitive decline [292, 293, 294], reinforcing the concept that lipid dysregulation intersects with core pathways in AD progression. In line with this, our preliminary data indicate that circulating LPC 18:2 declines in aged AD mice (unpublished) coincident with rising peripheral neutrophil counts, consistent with a lipid–immune imbalance that may amplify early neuroinflammation and synaptic dysfunction in vivo [232].

A key recent methodological advance has been the development of high‐resolution morphometric tools to quantify murine microglial phenotypes in situ. Using machine‐learning–assisted segmentation of Iba1^+^ microglia with masks capturing process architecture, these approaches distinguish reactive versus homeostatic morphologies with increased sensitivity [295]. Applied to 5xFAD mice, this analysis revealed evidence of early, sex‐biased microglial remodeling before overt plaque deposition. At very early ages (∼1.5–2 months), quantitative morphometry showed that female 5xFAD microglia had already begun to adopt reactive, amoeboid features (retracted/thickened processes and enlarged soma) compared with age‐matched males, particularly in hippocampal regions, despite minimal classical gliosis. By contrast, male 5xFAD microglia remained more hyper‐ramified at the same stage, and wild‐type mice of either sex did not show these shifts [295]. These findings support the possibility that female microglia enter a “primed‐to‐reactive” transition earlier, potentially contributing to sex‐biased trajectories of amyloid pathology and cognitive decline. Notably, the 5xFAD transgene is driven by a Thy1 promoter containing an estrogen response element, which may amplify Aβ expression in females [296] and should be explicitly considered when interpreting sex effects in this model.

Despite growing evidence that immune activation contributes to AD from early stages, reliable biomarkers of early neuroimmune remodeling remain limited. Canonical AD biomarkers (Aβ and tau) reflect proteinopathy but do not directly report neuroimmune state and can precede symptoms by years without indicating inflammatory activity [297, 298]. TSPO PET imaging has enabled in vivo glial monitoring but has recognized limitations, including low signal‐to‐noise for first‐generation ligands (e.g., [^11^C]PK11195), genetic binding variability for newer tracers (e.g., TSPO Ala147Thr), and limited functional specificity because TSPO is expressed in multiple glial populations and does not discriminate among microglial states [299, 300]. Moreover, some disease‐associated microglial programs may not upregulate TSPO, raising the possibility that TSPO‐based readouts miss key early activation states [300]. Peripheral inflammatory markers show inconsistent predictive value, and CSF measures (e.g., soluble TREM2) require invasive sampling. Together, these limitations highlight an unmet need for minimally invasive biomarkers that sensitively track early microglial remodeling in prodromal AD.

Looking ahead, a critical next step is to test whether quantitative features of microglial remodeling (e.g., branching complexity, soma hypertrophy, CD68‐associated phagolysosomal activity) correlate with distinct plasma metabolomic profiles, particularly bioactive lipid species such as LPC 18:2. Establishing these relationships will help resolve whether peripheral lipid changes merely track, or potentially precede, early neuroimmune transitions. Future studies should also evaluate whether composite lipid–immune signatures, combining lipid mediators with neutrophil phenotypes and other innate immune readouts, improve prediction of emerging microglial activation relative to any single measure. Importantly, the sex‐biased microglial patterns observed in 5xFAD mice raise the possibility that biomarker thresholds and trajectories differ between males and females, motivating explicit sex‐stratified testing in both preclinical models and longitudinal human cohorts.

Finally, dissecting mechanistic links among LPC biology, neutrophil persistence, and microglial remodeling remains an important frontier. It remains unresolved whether LPC 18:2 loss facilitates neutrophil‐mediated inflammatory signaling that secondarily shapes microglial behavior, or whether both reflect broader systemic metabolic dysregulation. Addressing these questions will require integrated lipidomic, immunophenotypic, and spatial microglial analyses across time, ideally in models that capture human‐relevant risk factors such as APOE‐ε4. Together, these lines of work support a broader paradigm in which neurodegeneration is approached not solely through protein aggregation, but through immunometabolic resolution, cellular communication, and sex‐specific neuroimmune resilience.

## 6 Sex Matters in Neuroinflammation: Translational Barriers and Pathways Forward

A review of the biomedical literature spanning the last four decades reported that only ~12% of human studies discussed sex as a biological variable [301]. Historically, both neuroscience and immunology often under‐emphasized sex in study design and analysis [302, 303]. Male animals became the default experimental model under assumptions of “convenience and control,” while female hormonal cycling was frequently treated as an unwieldy source of variability. Early observations of estrous‐cycle–linked behavioral variation contributed to the perception, often incorrectly generalized, that females were inherently more variable [304]. Practical and institutional factors reinforced this bias: female rodents were commonly reserved for breeding and were more costly, leading many laboratories to rely on surplus males [302]. Regulatory history also played a role. While policies from the 1990s onward required inclusion of females in US human clinical trials [305], comparable mandates for sex‐balanced design in preclinical animal research emerged much later, allowing male‐only paradigms to persist.

The cumulative impact has been a major sex‐based data gap. One widely cited analysis reported a ~5.5:1 male‐to‐female bias in rodent studies, with > 80% excluding females; even among neuroscience papers that included both sexes, only ~15% formally tested for sex effects [302]. These long‐standing design choices have entrenched blind spots in female neurobiology and limited mechanistic generalization across sexes. Many foundational mechanisms in immune and metabolic regulation were characterized largely in male animals, leaving critical questions unresolved regarding female‐specific pathways and sex‐by‐environment interactions. Although funders and journals increasingly require sex‐disaggregated reporting [306], the legacy of male‐centric design remains embedded throughout the neuroinflammation literature. Sex differences in murine neurological disease models are summarized in Table 2.

**TABLE 2:** Summary of sex differences in mouse models of human neurological disease.

Disease model	Mouse model name	Mouse model makeup	Disease pathology	Sex differences observed
Alzheimer's disease (AD)	APP/PS1	Human APP (Swedish) + PS1 mutation	Extracellular Aβ plaque deposition, gliosis	Increased expression of microglial activation genes in females (PMID: 34112929) Glycolytic, less phagocytic microglia in females associated with increased amyloidosis; amoeboid microglia in males (PMID: 34112929) Higher parenchymal Aβ burden, more severe cerebral amyloid angiopathy and subsequent microhemorrhage, higher levels of phosphorylated tau and proinflammatory cytokines, more severe astrocytosis and microgliosis, and greater neuronal and synaptic degenerations in females (PMID: 26707129) Greater amyloid burden and higher plaque number in females; greater Aβ40 and Aβ42 in age‐matched females (PMID: 14678749) Shorter female lifespan (PMID: 40138433)
	5xFAD	3 APP mutations (Swedish, Florida, London) + 2 PS1 mutations	Very early, aggressive Aβ plaque pathology, synaptic degeneration	Higher levels of Aβ42 and steady‐state transgenic APP in females; heightened inflammation in females (PMID: 25567526, PMID: 34864660) Greater amyloid deposition in the hippocampus and entorhinal cortex of females (PMID: 36899916) Sex‐based differences in cognitive performance (PMID: 34864660) Hyper‐ramified morphology in age‐matched male microglia; greater density and more reactive phenotype in female microglia (doi:10.1101/2025.10.07.681006)
	Tg2576	APP Swedish mutation	Age‐dependent Aβ plaque accumulation	Greater Aβ plaque load and occupation in age‐matched females (PMID: 11238065) Increased cognitive impairment in females (PMID: 30458163)
	3xTg‐AD	APP (Swedish) + PS1 + Tau (P301L)	Both Aβ plaques and tau neurofibrillary tangles	Greater Aβ burden and larger behavioral deficits in age‐matched females (PMID: 20934413) More aggressive Aβ pathology, increased beta‐secretase activity, and reduction of neprilysin in females (PMID: 18486110) More prominent amyloid plaques, neurofibrillary tangles, neuroinflammation and spatial cognitive deficits in females than in male mice (PMID: 30099679) Age‐dependent worsening of cognitive performance in females (PMID: 17659878)
	EFAD	5xFAD × human ApoE2/E3/E4	ApoE‐isoform–dependent modulation of Aβ burden and neuroinflammation	Increased cerebral cortex microbleeds in females (APOE4) (PMID: 26686669) Lower Iba1 plaque coverage and lower plaque compaction in females (APOE3) (PMID: 31113487) Lower TREM2 load (%Iba1) and lower TREM2 plaque coverage ratio in females (APOE3); fewer number of processes per microglia in the near plaque environment in females (APOE3) (PMID: 31113487)
	App^NL‐F^	APP knock‐in with Swedish (NL) and Iberian (F) mutations; normal APP expression levels	Progressive Aβ42 increase, plaques, gliosis, and mild cognitive deficits	Worse Morris water maze (MWM) performance in males compared to females (PMID: 34897085) Higher female minimal longevity but lower female maximal longevity (PMID: 40138433) Age‐dependent increased plaque burden in females (PMID: 40138433) Sex‐specific differences in memory deficits (PMID: 39543985)
	P301S/PS19 tau transgenic mice	Overexpression of human P301S mutant tau under the Prnp promoter	Tau accumulation and tangles with neurodegeneration, motor decline, and cognitive impairment	Earlier sleep disruption in females; chronic sleep disruption‐based spatial memory issues in males (PMID: 38858068) Sex‐based differences in behavior and phenotype; differential cytokine profiles by sex (PMID: 32093751)
Parkinson's disease (PD)	MPTP	Dopaminergic neurotoxin	Selective degeneration of substantia nigra dopaminergic neurons	Stride length decreased in males (PMID: 20347863) Decrease of dopamine, 3,4‐dihydroxyphenylacetic acid (DOPAC), and homovanillic acid (HVA) more pronounced in females (PMID: 19631714) More pronounced striatal and midbrain tyrosine hydroxylase (TH) protein decrease in females; increase in striatal glial fibrillary acidic protein (GFAP) in females; greater decrease of striatal dopamine transporter (DAT) protein in females (PMID: 19631714)
	6‐OHDA	Dopamine neurotoxin (lesion model)	Targeted loss of nigrostriatal dopaminergic projections	Lower dopaminergic cell loss and higher behavioral recovery in female rats (PMID: 15698888, PMID: 12535954) Sex‐specific responses to amphetamine and apomorphine in rats (PMID: 40389167) Early non‐motor impairments present in male but not female mice (PMID: 34710535) Increased oxidative stress in male mesencephalic neurons (PMID: 20416276)
	A53T α‐synuclein	Human α‐synuclein with A53T mutation (transgenic)	α‐synuclein aggregation and motor deficits	Sex‐specific differences in motor and memory tests (PMID: 32477098) Anxiety‐ and depression‐like behavior in males; reduction in alcohol drinking in males (PMID: 38284431) Sex‐ and age‐specific changes in brain ceramide metabolism (PMID: 38284431) Sex‐specific differences in weight gain, anxiety‐like behavior, and oxidative stress after high‐fat diet consumption (PMID: 40602652)
Post‐COVID‐19	SARS‐CoV‐2 K18‐hACE2	Human ACE2 under K18 promoter; infected with live SARS‐CoV‐2	Severe viral pneumonia and alveolar lung damage; viral dissemination including brain	Lower susceptibility to infection and less serious disease in females; higher ACE2 expression in the lung (PMID: 37609639, PMID: 37259182, PMCID: PMC9665217) Increased lung immune cell infiltration, greater tissue damage, and greater alveolar space reduction in males (PMCID: PMC9665217) Differential pulmonary infiltration of immune cells and cytokine profile by sex depending on SARS‐CoV‐2 variant (PMID: 40686017) Upregulation of soluble thrombomodulin (sTM) in females; increase in von Willebrand Factor (VWF) in males (PMCID: PMC9665217)
Traumatic brain injury (TBI)	Controlled cortical impact (CCI)	Mechanical cortical impact	Focal cortical contusion and neuronal loss	Improved motor performance in male mice and worse motor performance in female mice following progesterone treatment after CCI (PMID: 25280093) Sex‐specific differences in gut microbiota composition following CCI (PMID: 38410824) Greater astrocytic hypertrophy and heme‐oxygenase‐1 (HO‐1) induction in female rats post‐CCI, while males had increased endothelial activation and expression of beta‐catenin; increase in the number of vessels and complexity in males compared to females (PMID: 29648973) Sex‐based differential microglial and macrophage phenotype post‐CCI (PMID: 28608978)
	Fluid percussion injury (FPI)	Dural fluid pulse	Diffuse axonal injury	More robust defensive behavior to white noise in females following FPI (PMID: 33324313) Estrogen administration prior to FPI protective in male rats but exacerbates brain injury in female rats (PMID: 849535) Smaller contusion volume in female rats and greater numbers of NeuN‐positive cortical neurons in female mice (PMID: 14769396) Posttraumatic hypothermia reduced contusion volume in male but not female mice and protected male but not female mice against loss of cortical neurons (PMID: 14769396)
Multiple sclerosis (MS)	EAE (MOG/PLP/MBP)	Autoimmune demyelination	Immune‐mediated CNS demyelination and paralysis	Greater spinal cord infiltrating cells and demyelination in females; increased inflammatory and regulatory cell types in males (PMID: 33190849) Sex differences in measures of spinal cord inflammation and plasticity (PMID: 30649100) Greater EAE severity in SJL and ASW females; greater EAE incidence in NZW females; more severe disease in B10.PL and PL/J males (PMID: 15081249) Behavioral signs of neuropathic pain and disruption of the estrous cycle in females (PMID: 24581045)
	Cuprizone	Copper chelator diet	Toxic demyelination (especially corpus callosum)	Disruption of female estrous cycle (PMID: 19746424) More severe motor coordination deficits in males in the horizontal bar and passive wire hang tests; moe severe motor coordiantion deficits in females in the motor skill sequence test (PMID: 36552147) More anxiety‐like behaviors in males in the elevated zero maze (PMID: 36552147) More severe demyelination in SJL mice (PMID: 19016742)
Amyotrophic lateral sclerosis (ALS)	SOD1‐G93A	Mutant SOD1	Progressive motor neuron degeneration	Longer survival of females with SJL background and hybrid B6/SJL background; delayed disease onset in female mice with the C57BL/6 background (PMID: 16024047, PMID: 26594635) Sex‐based differences in the mitochondrial intermembrane space mitochondrial unfoldeed protein response (MS‐UPR^mt^) (PMID: 28186560)
	TDP‐43	Mutant/overexpressed TDP‐43	TDP‐43 proteinopathy with motor neuron loss	Age‐dependent onset of motor symptoms more pronounced in male mice with human WT TDP‐43 overexpression (PMID: 40847737) Impaired electroretinogram (ERG) responses in retinas of young female TDP‐43^M337V^ mice; sex‐specific differences in key regulators of mitochondrial dynamics and bioenergetics (PMID: 38143367)

### 6.1 Roadblocks to Studying Sex Bias in Neuroinflammatory Disease

Despite growing awareness, structural and analytical barriers continue to limit progress in dissecting the effects of sex as a biological variable. Many cohort studies and clinical trials remain unstratified by sex or underpowered to detect sex effects, and large omics initiatives often lack sex‐balanced sampling [302, 307]. Analytical bias persists even when both sexes are included. One evaluation found that among clinical studies enrolling both sexes, fewer than half (42%) conducted or reported sex‐disaggregated analyses [308]. Similarly, surveys suggest that while many experiments now include both sexes, fewer than ~15% statistically analyze sex as a factor [309]. As a result, sex‐specific cellular programs, particularly in CNS‐resident immune populations, can be missed when data are pooled.

Confounding biological variables further complicate interpretation. Hormonal transitions (e.g., menopause), metabolic comorbidities (e.g., obesity and metabolic syndrome), and aging can interact with neuroinflammatory disease in sex‐specific ways [310, 311, 312], yet these interactions are often not modeled explicitly. On the translational side, sex‐agnostic reporting remains common even in pivotal therapeutic trials. In AD, re‐analyses of recent trials (e.g., lecanemab) have raised the possibility of sex‐dependent efficacy signals [313, 314], but most studies were not designed or powered to test sex‐specific outcomes. Without systematic incorporation of sex into trial design, power calculations, and analysis plans, including sex‐by‐age interaction models and careful adjustment for key confounders, true biological differences may remain obscured.

### 6.2 Limitations of Current Animal Models

Animal models aim to capture key features of human immune–metabolic neuroinflammatory disease (summarized in Table 2). Yet rodent immune systems and metabolic programs differ from humans in meaningful ways [315, 316, 317]. For example, while activated microglia in both mice and humans shift toward glycolysis, species can rely on different rate‐limiting enzymes and regulatory nodes (e.g., hexokinase‐centered programs in mice versus phosphofructokinase‐linked programs in human myeloid cells), underscoring nontrivial divergence in immunometabolic control points [318]. Rodents also do not model human endocrine aging trajectories well (e.g., menstruation, menopause, multi‐decade hormonal change) [319], limiting interpretability of sex effects across the human lifespan.

Moreover, commonly used transgenic and toxin‐based models often isolate a single pathological feature and compress chronic disease into accelerated timelines, for example, amyloid plaque focused models in AD, toxin‐induced dopaminergic loss in PD, or chemically induced autoimmunity in MS. These approaches can underrepresent gradual progression, mixed pathology, and interactions with systemic metabolism that are central to human disease. Persisting male bias in preclinical work further compounds these limitations. The thalidomide tragedy remains a canonical reminder of how sex‐ and pregnancy‐linked risks can be missed when safety evaluation fails to model relevant biology [320]. Together, limited biological fidelity and sex imbalance in experimental systems represent a major translational bottleneck for discovery and therapeutic development in neuroinflammatory disease.

### 6.3 Toward Next‐Generation Models and Study Designs

Future models must deliberately incorporate sex, age, and metabolic context. Preclinical studies should include both sexes across appropriate life stages and explicitly consider hormonal status (e.g., modeling menopause/senescence in females alongside comparable aging in males). Aged cohorts are essential. Genetic diversity should also be embraced to better mirror human heterogeneity. The AD‐BXD panel provides an instructive example. The BXD recombinant inbred strains, derived from C57BL/6J and DBA/2 J backgrounds, capture extensive natural genetic variation and enable mapping of modifiers of neurodegeneration and immunity. Introducing AD transgenes (e.g., 5xFAD or APP/PS1) into genetically diverse BXD lines yields stable strains that share a pathogenic driver but differ across thousands of naturally segregating alleles. This strategy reveals strain‐dependent differences in amyloid deposition, neurodegeneration, microglial activation, synaptic integrity, and cognitive decline [321, 322], including resilience in some strains despite the same initiating mutation [322]. Such trajectories map onto genetic modifiers enriched for immune, metabolic, and lipid‐handling pathways, strengthening the bridge between mechanistic neurobiology and human risk architecture.

Ideal future models will also incorporate human risk alleles (e.g., APOE variants), endocrine‐genetic factors (including variation in sex hormone receptors), and environmental modulators (dietary fat, microbiome composition) that reshape metabolism. These systems should be deeply phenotyped with multi‐omic approaches, paired single‐cell transcriptomics, proteomics, metabolomics/lipidomics, and immune profiling in CNS tissues to define sex‐specific pathways. Animal‐derived pathway signatures should be validated in human brain tissue and biofluids to ensure translational relevance.

## 7 Conclusion

**FIGURE 1:**
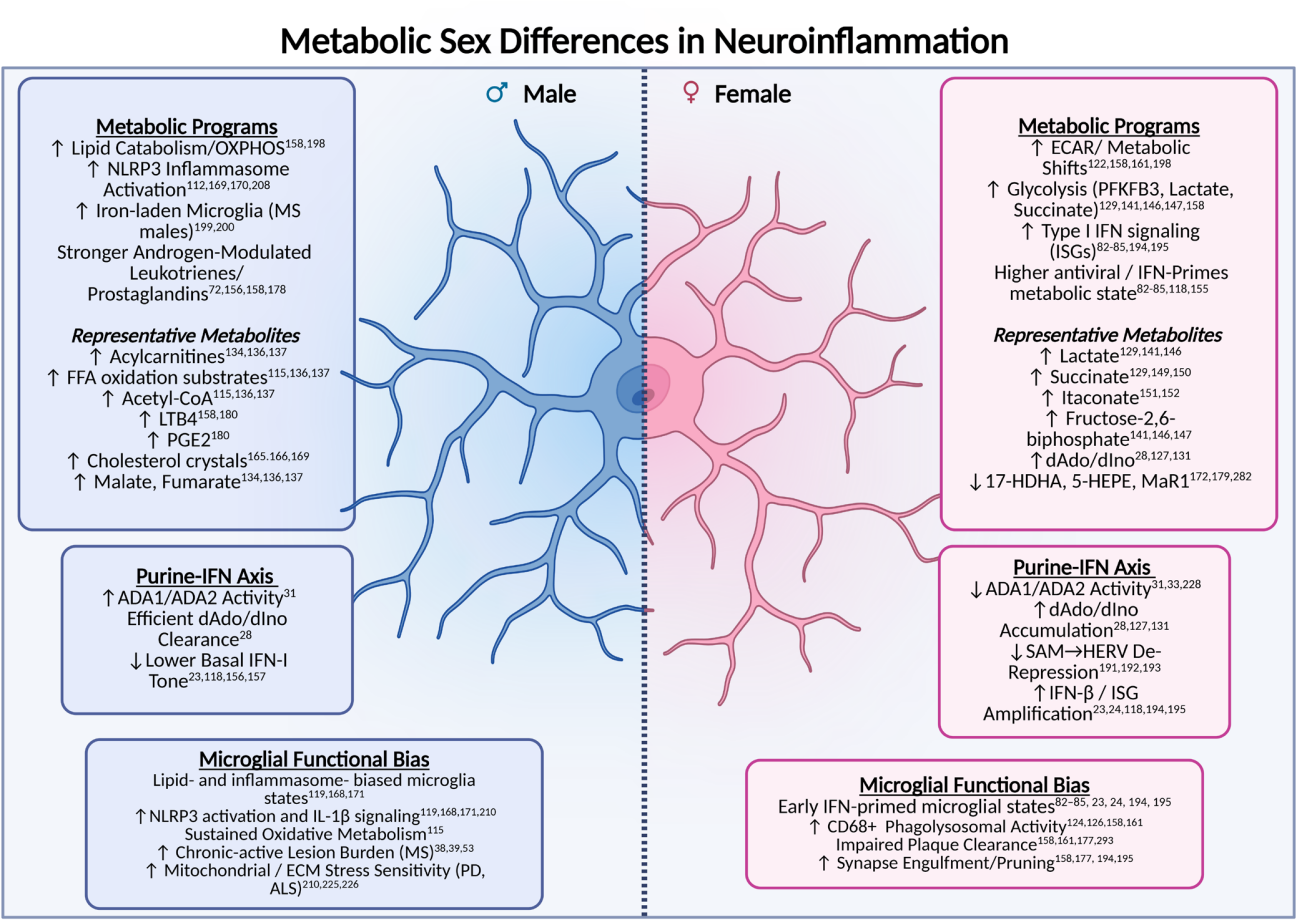
Sex‐biased immunometabolic programs shape microglial IFN signaling and neuroinflammatory vulnerability. This figure summarizes sex‐associated differences in microglial immunometabolic programs that shape innate immune signaling and neuroinflammatory outcomes across neurodegenerative and neuroinflammatory diseases. Male‐ and female‐associated features are shown as enriched tendencies across models and disease contexts, rather than mutually exclusive states. Male‐biased microglial programs are characterized by increased reliance on lipid catabolism and oxidative phosphorylation (OXPHOS), enhanced inflammasome‐associated signaling, and androgen‐modulated eicosanoid pathways. Higher ADA1/ADA2 activity promotes efficient purine clearance and is associated with a lower basal type I interferon (IFN‐I) tone. Functionally, these programs bias microglia toward lipid‐ and inflammasome‐driven inflammatory states, increased chronic lesion burden, and heightened sensitivity to mitochondrial and oxidative stress. Female‐biased microglial programs preferentially engage glycolytic and ECAR‐high metabolic states, coupled to stronger IFN‐responsive transcriptional programs. Reduced ADA1/ADA2 activity permits purine accumulation, facilitating SAM‐dependent derepression of endogenous retroelements and amplification of IFN‐β and interferon‐stimulated gene expression. These states are associated with early IFN priming, increased phagolysosomal activity, impaired plaque clearance, and enhanced synaptic engulfment or pruning. Together, these sex‐biased immunometabolic programs converge on the purine‐interferon axis, providing a conceptual framework for how biological sex modulates microglial IFN tone, inflammatory trajectories, and susceptibility to neurodegenerative pathology.

Across this review, metabolic–immune axes emerge as central to sex‐biased neuroinflammatory vulnerability (Figure 1). Purine metabolism and adenosine signaling tune CNS immunity and intersect with sex‐biased interferon set points. Activated glia undergo glycolytic remodeling during inflammation, yet key control nodes can differ by sex and species. Lipid sensing pathways (including PPAR/LXR‐linked programs) shape microglial activation in sex‐dependent ways, consistent with known sex differences in lipid metabolism. The cGAS–STING–interferon axis exemplifies an immune–metabolic node with functional sex divergence: in one model, activating STING in spinal microglia relieved neuropathic pain in male mice but elicited counterproductive inflammatory responses in females [323]. Together, these circuits align with clinical patterns in which female‐skewed conditions (e.g., AD, MS) often feature prominent inflammatory and interferon‐linked signatures, while male‐skewed disorders (e.g., PD, ALS) exhibit distinct metabolic vulnerabilities.

Progress now requires treating sex and metabolic state as core variables in study design, analysis, and translation. This includes powering cohorts for sex‐stratified inference; modeling sex‐by‐age and sex‐by‐metabolism interactions; building and profiling sex‐balanced, metabolically informed experimental systems; and developing therapies that target glycolysis, purine turnover, lipid signaling, and interferon pathways in sex‐aware ways. Only through a sex‐specific, immunometabolically grounded approach can the field uncover actionable mechanisms and develop effective interventions for both females and males.

## Funding

The work discussed in this review was sponsored by generous donation of philanthropic funds from the following sources: The Clouse Family, The Shiley Foundation, The Shirley and Harry Beer Charitable Foundation, Nancy Vaughan, Nabil and Gayda Hanna, Brad and Rachel Greenwald, Bridget Cresto, James and Katya Hazel, the Prebys Foundation, and CZI grant DAF2019‐198153 from the Chan Zuckerberg Initiative DAF, and an advised fund of Silicon Valley Community Foundation. This work was also supported by National Institutes of Health (NIH) Grant numbers: R01CA273230 (S.S. and P.S) and U54AG065141 (S.S and P.S). AG was supported by the BioLegend Fellowship in Immunology.

## Conflicts of Interest

The authors declare no conflicts of interest.

## Data Availability

All data used in the preparation of the article will be made available by the corresponding author.

## References

[1] F.‐D. Shi and V. W. Yong , “Neuroinflammation Across Neurological Diseases,” Science 388 (2025): eadx0043. 40536983 10.1126/science.adx0043

[2] R. M. Ransohoff , “How Neuroinflammation Contributes to Neurodegeneration,” Science 353 (2016): 777–783. 27540165 10.1126/science.aag2590

[3] J. Leffler , S. Trend , S. Gorman , and P. H. Hart , “Sex‐Specific Environmental Impacts on Initiation and Progression of Multiple Sclerosis,” Frontiers in Neurology 13 (2022): 835162. 35185777 10.3389/fneur.2022.835162 PMC8850837

[4] P. Bede , M. Elamin , S. Byrne , and O. Hardiman , “Sexual Dimorphism in ALS: Exploring Gender‐Specific Neuroimaging Signatures,” Amyotrophic Lateral Sclerosis and Frontotemporal Degeneration 15 (2014): 235–243. 24344910 10.3109/21678421.2013.865749

[5] A. Canosa , A. Martino , U. Manera , et al., “Sex‐Related Differences in Amyotrophic Lateral Sclerosis: A 2‐[18F]FDG‐PET Study,” European Journal of Neurology 32 (2025): e16588. 39655539 10.1111/ene.16588 PMC11629101

[6] L. Del Hoyo Soriano , O. Wagemann , A. Bejanin , J. Levin , and J. Fortea , “Sex‐Related Differences in Genetically Determined Alzheimer's Disease,” Frontiers in Aging Neuroscience 17 (2025): 1522434. 40103931 10.3389/fnagi.2025.1522434 PMC11913828

[7] C. Lopez‐Lee , E. R. S. Torres , G. Carling , and L. Gan , “Mechanisms of Sex Differences in Alzheimer's Disease,” Neuron 112 (2024): 1208–1221. 38402606 10.1016/j.neuron.2024.01.024 PMC11076015

[8] J. C. Jurado‐Coronel , R. Cabezas , M. F. Ávila Rodríguez , V. Echeverria , L. M. García‐Segura , and G. E. Barreto , “Sex Differences in Parkinson's Disease: Features on Clinical Symptoms, Treatment Outcome, Sexual Hormones and Genetics,” Frontiers in Neuroendocrinology 50 (2018): 18–30. 28974386 10.1016/j.yfrne.2017.09.002

[9] S. M. Gold , A. Willing , F. Leypoldt , F. Paul , and M. A. Friese , “Sex Differences in Autoimmune Disorders of the Central Nervous System,” Seminars in Immunopathology 41 (2019): 177–188. 30361800 10.1007/s00281-018-0723-8

[10] C. A. Colvert and M. A. Cunningham , “Editorial: Sexual Dimorphism in Autoimmune and Immune‐Dysregulated Diseases,” Frontiers in Immunology 15 (2024): 1531757. 39712024 10.3389/fimmu.2024.1531757 PMC11659246

[11] K. M. Rexrode , T. E. Madsen , A. Y. X. Yu , C. Carcel , J. H. Lichtman , and E. C. Miller , “The Impact of Sex and Gender on Stroke,” Circulation Research 130 (2022): 512–528. 35175851 10.1161/CIRCRESAHA.121.319915 PMC8890686

[12] H. Abdu and G. Seyoum , “Sex Differences in Stroke Risk Factors, Clinical Profiles, and In‐Hospital Outcomes Among Stroke Patients Admitted to the Medical Ward of Dessie Comprehensive Specialized Hospital, Northeast Ethiopia,” Degenerative Neurological and Neuromuscular Diseases 12 (2022): 133–144. 10.2147/DNND.S383564 PMC9595065 36304698

[13] R. Gupte , W. Brooks , R. Vukas , J. Pierce , and J. Harris , “Sex Differences in Traumatic Brain Injury: What We Know and What We Should Know,” Journal of Neurotrauma 36 (2019): 3063–3091. 30794028 10.1089/neu.2018.6171 PMC6818488

[14] D. P. Shah , T. Thaweethai , E. W. Karlson , et al., “Sex Differences in Long COVID,” JAMA Network Open 8 (2025): e2455430. 39841477 10.1001/jamanetworkopen.2024.55430 PMC11755195

[15] E. Ortona and W. Malorni , “Long COVID: To Investigate Immunological Mechanisms and Sex/Gender Related Aspects as Fundamental Steps for Tailored Therapy,” European Respiratory Journal 59 (2022): 2102245. 34531277 10.1183/13993003.02245-2021 PMC8462012

[16] A. Villa , P. Gelosa , L. Castiglioni , et al., “Sex‐Specific Features of Microglia From Adult Mice,” Cell Reports 23 (2018): 3501–3511. 29924994 10.1016/j.celrep.2018.05.048 PMC6024879

[17] S. R. Ocañas , V. A. Ansere , C. M. Kellogg , J. V. V. Isola , A. J. Chucair‐Elliott , and W. M. Freeman , “Chromosomal and Gonadal Factors Regulate Microglial Sex Effects in the Aging Brain,” Brain Research Bulletin 195 (2023): 157–171. 36804773 10.1016/j.brainresbull.2023.02.008 PMC10810555

[18] B. Angeloni , R. Bigi , G. Bellucci , et al., “A Case of Double Standard: Sex Differences in Multiple Sclerosis Risk Factors,” International Journal of Molecular Sciences 22 (2021): 3696. 33918133 10.3390/ijms22073696 PMC8037645

[19] S. Cerri , L. Mus , and F. Blandini , “Parkinson's Disease in Women and Men: What's the Difference?,” Journal of Parkinson's Disease 9 (2019): 501–515. 10.3233/JPD-191683 PMC6700650 31282427

[20] L. Müller , S. Di Benedetto , and V. Müller , “Influence of Biological Sex on Neuroinflammatory Dynamics in the Aging Brain,” Frontiers in Aging Neuroscience 17 (2025): 1670175. 40951920 10.3389/fnagi.2025.1670175 PMC12426254

[21] J. A. Chowen , P. Argente‐Arizón , A. Freire‐Regatillo , and J. Argente , “Sex Differences in the Neuroendocrine Control of Metabolism and the Implication of Astrocytes,” Frontiers in Neuroendocrinology 48 (2018): 3–12. 28552663 10.1016/j.yfrne.2017.05.003

[22] M. Caldarelli , P. Rio , A. Marrone , et al., “Gut‐Brain Axis: Focus on Sex Differences in Neuroinflammation,” International Journal of Molecular Sciences 25 (2024): 5377. 38791415 10.3390/ijms25105377 PMC11120930

[23] T. Goldmann , T. Blank , and M. Prinz , “Fine‐Tuning of Type I IFN‐Signaling in Microglia—Implications for Homeostasis, CNS Autoimmunity and Interferonopathies,” Current Opinion in Neurobiology 36 (2016): 38–42. 26397019 10.1016/j.conb.2015.09.003 PMC7126514

[24] C. C. Escoubas , L. C. Dorman , P. T. Nguyen , et al., “Type‐I‐Interferon‐Responsive Microglia Shape Cortical Development and Behavior,” Cell 187 (2024): 1936–1954.e24. 38490196 10.1016/j.cell.2024.02.020 PMC11015974

[25] P. Prakash , C. E. Randolph , K. A. Walker , and G. Chopra , “Lipids: Emerging Players of Microglial Biology,” Glia 73 (2025): 657–677. 39688320 10.1002/glia.24654 PMC11784843

[26] Y. Kushnareva , I. T. Mathews , A. Y. Andreyev , et al., “Functional Analysis of Immune Signature Genes in Th1* Memory Cells Links ISOC1 and Pyrimidine Metabolism to IFN‐γ and IL‐17 Production,” Journal of Immunology (Baltimore, Md.: 1950) 206 (2021): 1181–1193. 33547171 10.4049/jimmunol.2000672 PMC7946769

[27] M. Takahashi , C. J. Lio , A. Campeau , et al., “The Tumor Suppressor Kinase DAPK3 Drives Tumor‐Intrinsic Immunity Through the STING‐IFN‐β Pathway,” Nature Immunology 22 (2021): 485–496. 33767426 10.1038/s41590-021-00896-3 PMC8300883

[28] R. Dhanwani , M. Takahashi , I. T. Mathews , et al., “Cellular Sensing of Extracellular Purine Nucleosides Triggers an Innate IFN‐β Response,” Science Advances 6 (2020): eaba3688. 32743071 10.1126/sciadv.aba3688 PMC7375821

[29] X. Hu , H. Zhang , Q. Zhang , X. Yao , W. Ni , and K. Zhou , “Emerging Role of STING Signalling in CNS Injury: Inflammation, Autophagy, Necroptosis, Ferroptosis and Pyroptosis,” Journal of Neuroinflammation 19 (2022): 242. 36195926 10.1186/s12974-022-02602-y PMC9531511

[30] A. Nazmi , R. H. Field , E. W. Griffin , et al., “Chronic Neurodegeneration Induces Type I Interferon Synthesis via STING, Shaping Microglial Phenotype and Accelerating Disease Progression,” Glia 67 (2019): 1254–1276. 30680794 10.1002/glia.23592 PMC6520218

[31] I. Meyts and I. Aksentijevich , “Deficiency of Adenosine Deaminase 2 (DADA2): Updates on the Phenotype, Genetics, Pathogenesis, and Treatment,” Journal of Clinical Immunology 38 (2018): 569–578. 29951947 10.1007/s10875-018-0525-8 PMC6061100

[32] P. Saminathan , I. T. Mathews , A. Alimadadi , et al., “Sex Differences in Adenosine Deaminase Activity Associate With Disparities in SARS‐CoV‐2 Innate Immunity,” iScience 28 (2025): 112418. 40343269 10.1016/j.isci.2025.112418 PMC12059719

[33] K. S. Barron , I. Aksentijevich , N. T. Deuitch , et al., “The Spectrum of the Deficiency of Adenosine Deaminase 2: An Observational Analysis of a 60 Patient Cohort,” Frontiers in Immunology 12 (2021): 811473. 35095905 10.3389/fimmu.2021.811473 PMC8790931

[34] S. Sharma , A. Gibbons , and E. O. Saphire , “Sex Differences in Tissue‐Specific Immunity and Immunology,” Science 389 (2025): 599–603. 40773572 10.1126/science.adx4381 PMC12777860

[35] M. Corey , M. Rayadurgam , J. Jung , et al., “Deoxyguanosine Kinase Deficiency Couples Purine Metabolism to Innate Immune Activation and Lipid Accumulation in Hepatocytes,” bioRxiv: The Preprint Server for Biology (2025), 10.1101/2025.11.18.688341. PMC13416635 42529171

[36] J. Graf , M. K. Akmatov , S. G. Meuth , H. Tremlett , and J. Holstiege , “Updated Multiple Sclerosis Incidence, 2015‐2022,” JAMA Neurology 81 (2024): 1100–1102. 39250144 10.1001/jamaneurol.2024.2876 PMC11385316

[37] H. Kamali , S. Hosseini , H. R. Aliabadi , M. Poursadeghfard , M. A. Sahraian , and S. Eskandarieh , “Increasing Incidence and Male to Female Sex Ratio of Multiple Sclerosis in Tehran, Iran: A Population‐Based Study,” Iranian Journal of Public Health 51 (2022): 1193–1194. 36407743 10.18502/ijph.v51i5.9439 PMC9643219

[38] K. A. Ribbons , P. McElduff , C. Boz , et al., “Male Sex Is Independently Associated With Faster Disability Accumulation in Relapse‐Onset MS but Not in Primary Progressive MS,” PLoS One 10 (2015): e0122686. 26046348 10.1371/journal.pone.0122686 PMC4457630

[39] E. Crabtree‐Hartman , “Sex Differences in Multiple Sclerosis,” Continuum (Minneap. Minn.) 16 (2010): 193–210. 22810606 10.1212/01.CON.0000389942.81981.72

[40] J. Dalmau , E. Tüzün , H. Y. Wu , et al., “Paraneoplastic Anti‐N‐Methyl‐D‐Aspartate Receptor Encephalitis Associated With Ovarian Teratoma,” Annals of Neurology 61 (2007): 25–36. 17262855 10.1002/ana.21050 PMC2430743

[41] M. J. Titulaer , L. McCracken , I. Gabilondo , et al., “Treatment and Prognostic Factors for Long‐Term Outcome in Patients With Anti‐NMDA Receptor Encephalitis: An Observational Cohort Study,” Lancet Neurology 12 (2013): 157–165. 23290630 10.1016/S1474-4422(12)70310-1 PMC3563251

[42] Alzheimer's Association , “Alzheimer's Disease Facts and Figures,” Alzheimer's & Dementia 11 (2015): 332–384. 10.1016/j.jalz.2015.02.003 25984581

[43] D. Zhu , A. Montagne , and Z. Zhao , “Alzheimer's Pathogenic Mechanisms and Underlying Sex Difference,” Cellular and Molecular Life Sciences 78 (2021): 4907–4920. 33844047 10.1007/s00018-021-03830-w PMC8720296

[44] G. F. Wooten , L. J. Currie , V. E. Bovbjerg , J. K. Lee , and J. Patrie , “Are Men at Greater Risk for Parkinson's Disease Than Women?,” Journal of Neurology, Neurosurgery, and Psychiatry 75 (2004): 637–639. 15026515 10.1136/jnnp.2003.020982 PMC1739032

[45] C. A. Haaxma , B. R. Bloem , G. F. Borm , et al., “Gender Differences in Parkinson's Disease,” Journal of Neurology, Neurosurgery, and Psychiatry 78 (2007): 819–824. 17098842 10.1136/jnnp.2006.103788 PMC2117736

[46] P. A. McCombe and R. D. Henderson , “Effects of Gender in Amyotrophic Lateral Sclerosis,” Gender Medicine 7 (2010): 557–570. 21195356 10.1016/j.genm.2010.11.010

[47] A. Chiò , G. Mora , A. Calvo , et al., “Epidemiology of ALS in Italy: A 10‐Year Prospective Population‐Based Study,” Neurology 72 (2009): 725–731. 19237701 10.1212/01.wnl.0000343008.26874.d1

[48] J. H. Veldink , “What Does Age at Onset in ALS Tell Us About the Genetic Basis of the Disease?,” Journal of Neurology, Neurosurgery, and Psychiatry 90 (2019): 250. 30355608 10.1136/jnnp-2018-319473

[49] S. Seshadri , A. Beiser , M. Kelly‐Hayes , et al., “The Lifetime Risk of Stroke: Estimates From the Framingham Study: Estimates From the Framingham Study,” Stroke 37 (2006): 345–350. 16397184 10.1161/01.STR.0000199613.38911.b2

[50] L. Li , C. A. Scott , and P. M. Rothwell , “Association of Younger vs Older Ages With Changes in Incidence of Stroke and Other Vascular Events, 2002‐2018,” JAMA 328 (2022): 563–574. 35943470 10.1001/jama.2022.12759 PMC9364129

[51] E. Rodríguez‐Castro , M. Rodríguez‐Yáñez , S. Arias , et al., “Influence of Sex on Stroke Prognosis: A Demographic, Clinical, and Molecular Analysis,” Frontiers in Neurology 10 (2019): 388. 31057479 10.3389/fneur.2019.00388 PMC6478658

[52] C. L. Gibson , “Cerebral Ischemic Stroke: Is Gender Important?,” Journal of Cerebral Blood Flow and Metabolism 33 (2013): 1355–1361. 23756694 10.1038/jcbfm.2013.102 PMC3764377

[53] P. K. Coyle , “What Can We Learn From Sex Differences in MS?,” Journal of Personalized Medicine 11 (2021): 1006. 34683148 10.3390/jpm11101006 PMC8537319

[54] N. Miyaue and M. Nagai , “Sex Differences in the Pharmacokinetics of Levodopa and Carbidopa in Patients With Parkinson's Disease,” Parkinsonism & Related Disorders 139 (2025): 108006. 40845588 10.1016/j.parkreldis.2025.108006

[55] N. Shobha , P. N. Sylaja , M. K. Kapral , J. Fang , M. D. Hill , and Investigators of the Registry of the Canadian Stroke Network , “Differences in Stroke Outcome Based on Sex,” Neurology 74 (2010): 767–771. 20194917 10.1212/WNL.0b013e3181d5275c PMC2836873

[56] R. W. Persky , L. C. Turtzo , and L. D. McCullough , “Stroke in Women: Disparities and Outcomes,” Current Cardiology Reports 12 (2010): 6–13. 20425178 10.1007/s11886-009-0080-2 PMC2861793

[57] H. Joo , C. Gu , M. Wiest , et al., “Differential Expression of Nuclear Hormone Receptors by Dendritic Cell Subsets in Human Vaginal Mucosa and Skin,” Frontiers in Immunology 13 (2022): 1063343. 36713394 10.3389/fimmu.2022.1063343 PMC9880315

[58] N. Fuentes and P. Silveyra , “Estrogen Receptor Signaling Mechanisms,” Advances in Protein Chemistry and Structural Biology 116 (2019): 135–170. 31036290 10.1016/bs.apcsb.2019.01.001 PMC6533072

[59] G. Notas , M. Kampa , and E. Castanas , “G Protein‐Coupled Estrogen Receptor in Immune Cells and Its Role in Immune‐Related Diseases,” Front Endocrinol (Lausanne) 11 (2020): 579420. 33133022 10.3389/fendo.2020.579420 PMC7564022

[60] Y. Zhu , S. Duan , M. Wang , Z. Deng , and J. Li , “Neuroimmune Interaction: A Widespread Mutual Regulation and the Weapons for Barrier Organs,” Frontiers in Cell and Developmental Biology 10 (2022): 906755. 35646918 10.3389/fcell.2022.906755 PMC9130600

[61] S. S. Chavan , V. A. Pavlov , and K. J. Tracey , “Mechanisms and Therapeutic Relevance of Neuro‐Immune Communication,” Immunity 46 (2017): 927–942. 28636960 10.1016/j.immuni.2017.06.008 PMC5578398

[62] T. W. Hodo , M. T. P. de Aquino , A. Shimamoto , and A. Shanker , “Critical Neurotransmitters in the Neuroimmune Network,” Frontiers in Immunology 11 (2020): 1869. 32973771 10.3389/fimmu.2020.01869 PMC7472989

[63] G. Benedek , J. Zhang , S. Bodhankar , et al., “Estrogen Induces Multiple Regulatory B Cell Subtypes and Promotes M2 Microglia and Neuroprotection During Experimental Autoimmune Encephalomyelitis,” Journal of Neuroimmunology 293 (2016): 45–53. 27049561 10.1016/j.jneuroim.2016.02.009 PMC4824954

[64] S. Ghisletti , C. Meda , A. Maggi , and E. Vegeto , “17beta‐Estradiol Inhibits Inflammatory Gene Expression by Controlling NF‐kappaB Intracellular Localization,” Molecular and Cellular Biology 25 (2005): 2957–2968. 15798185 10.1128/MCB.25.8.2957-2968.2005 PMC1069609

[65] A. J. Murphy , P. M. Guyre , and P. A. Pioli , “Estradiol Suppresses NF‐Kappa B Activation Through Coordinated Regulation of Let‐7a and miR‐125b in Primary Human Macrophages,” Journal of Immunology 184 (2010): 5029–5037. 10.4049/jimmunol.0903463 PMC2882792 20351193

[66] V. Luine and M. Frankfurt , “Interactions Between Estradiol, BDNF and Dendritic Spines in Promoting Memory,” Neuroscience 239 (2013): 34–45. 23079626 10.1016/j.neuroscience.2012.10.019 PMC3597766

[67] J. L. Spencer , E. M. Waters , R. D. Romeo , G. E. Wood , T. A. Milner , and B. McEwen , “Uncovering the Mechanisms of Estrogen Effects on Hippocampal Function,” Frontiers in Neuroendocrinology 29 (2008): 219–237. 18078984 10.1016/j.yfrne.2007.08.006 PMC2440702

[68] J. W. Simpkins , K. D. Yi , S.‐H. Yang , and J. A. Dykens , “Mitochondrial Mechanisms of Estrogen Neuroprotection,” Biochimica et Biophysica Acta 1800 (2010): 1113–1120. 19931595 10.1016/j.bbagen.2009.11.013 PMC2889195

[69] H. Du , A. Mizokami , J. Ni , et al., “Role of Testosterone Signaling in Microglia: A Potential Role for Sex‐Related Differences in Alzheimer's Disease,” Advanced Science 12 (2025): e2413375. 40125707 10.1002/advs.202413375 PMC12097063

[70] L. Henze , N. Will , D. Lee , et al., “Testosterone Affects Female CD4+ T Cells in Healthy Individuals and Autoimmune Liver Diseases,” JCI Insight 10 (2025): e184544. 40260919 10.1172/jci.insight.184544 PMC12016935

[71] S. N. Alexander , A. R. Green , E. K. Debner , et al., “The Influence of Sex on Neuroimmune Communication, Pain, and Physiology,” Biology of Sex Differences 15 (2024): 82. 39439003 10.1186/s13293-024-00660-w PMC11494817

[72] A. Traish , J. Bolanos , S. Nair , F. Saad , and A. Morgentaler , “Do Androgens Modulate the Pathophysiological Pathways of Inflammation? Appraising the Contemporary Evidence,” Journal of Clinical Medicine 7 (2018): 549. 30558178 10.3390/jcm7120549 PMC6306858

[73] M. Fijak , E. Schneider , J. Klug , et al., “Testosterone Replacement Effectively Inhibits the Development of Experimental Autoimmune Orchitis in Rats: Evidence for a Direct Role of Testosterone on Regulatory T Cell Expansion,” Journal of Immunology 186 (2011): 5162–5172. 10.4049/jimmunol.1001958 21441459

[74] S. Altuwaijri , K.‐H. Chuang , K.‐P. Lai , et al., “Susceptibility to Autoimmunity and B Cell Resistance to Apoptosis in Mice Lacking Androgen Receptor in B Cells,” Molecular Endocrinology 23 (2009): 444–453. 19164450 10.1210/me.2008-0106 PMC2667704

[75] R. P. Singh and D. S. Bischoff , “Sex Hormones and Gender Influence the Expression of Markers of Regulatory T Cells in SLE Patients,” Frontiers in Immunology 12 (2021): 619268. 33746959 10.3389/fimmu.2021.619268 PMC7966510

[76] V. L. Kronzer , S. L. Bridges, Jr. , and J. M. Davis, 3rd , “Why Women Have More Autoimmune Diseases Than Men: An Evolutionary Perspective,” Evolutionary Applications 14 (2021): 629–633. 33767739 10.1111/eva.13167 PMC7980266

[77] S. L. Klein and K. L. Flanagan , “Sex Differences in Immune Responses,” Nature Reviews Immunology 16 (2016): 626–638. 10.1038/nri.2016.90 27546235

[78] T. Takahashi and A. Iwasaki , “Sex Differences in Immune Responses,” Science 371 (2021): 347–348. 33479140 10.1126/science.abe7199

[79] L. Carrel and H. F. Willard , “X‐Inactivation Profile Reveals Extensive Variability in X‐Linked Gene Expression in Females,” Nature 434 (2005): 400–404. 15772666 10.1038/nature03479

[80] M. Souyris , C. Cenac , P. Azar , et al., “TLR7 Escapes X Chromosome Inactivation in Immune Cells,” Science Immunology 3 (2018): eaap8855. 29374079 10.1126/sciimmunol.aap8855

[81] A. Youness , C. Cenac , B. Faz‐López , et al., “TLR8 Escapes X Chromosome Inactivation in Human Monocytes and CD4+ T Cells,” Biology of Sex Differences 14 (2023): 60. 37723501 10.1186/s13293-023-00544-5 PMC10506212

[82] K. Webb , H. Peckham , A. Radziszewska , et al., “Sex and Pubertal Differences in the Type 1 Interferon Pathway Associate With Both X Chromosome Number and Serum Sex Hormone Concentration,” Frontiers in Immunology 9 (2018): 3167. 30705679 10.3389/fimmu.2018.03167 PMC6345344

[83] M. Griesbeck , S. Ziegler , S. Laffont , et al., “Sex Differences in Plasmacytoid Dendritic Cell Levels of IRF5 Drive Higher IFN‐α Production in Women,” Journal of Immunology (Baltimore, Md.: 1950) 195 (2015): 5327–5336. 26519527 10.4049/jimmunol.1501684 PMC4654231

[84] B. Berghöfer , T. Frommer , G. Haley , L. Fink , G. Bein , and H. Hackstein , “TLR7 Ligands Induce Higher IFN‐Alpha Production in Females,” Journal of Immunology (Baltimore, Md.: 1950) 177 (2006): 2088–2096. 16887967 10.4049/jimmunol.177.4.2088

[85] T. Wang , J. Marken , J. Chen , et al., “High TLR7 Expression Drives the Expansion of CD19+CD24hiCD38hi Transitional B Cells and Autoantibody Production in SLE Patients,” Frontiers in Immunology 10 (2019): 1243. 31231380 10.3389/fimmu.2019.01243 PMC6559307

[86] E. J. Pone , Z. Xu , C. A. White , H. Zan , and P. Casali , “B Cell TLRs and Induction of Immunoglobulin Class‐Switch DNA Recombination,” Front Biosci (Landmark Ed) 17 (2012): 2594–2615. 22652800 10.2741/4073 PMC4095906

[87] G. J. Brown , P. F. Cañete , H. Wang , et al., “TLR7 Gain‐Of‐Function Genetic Variation Causes Human Lupus,” Nature 605 (2022): 349–356. 35477763 10.1038/s41586-022-04642-z PMC9095492

[88] S. Squillace and D. Salvemini , “Toll‐Like Receptor‐Mediated Neuroinflammation: Relevance for Cognitive Dysfunctions,” Trends in Pharmacological Sciences 43 (2022): 726–739. 35753845 10.1016/j.tips.2022.05.004 PMC9378500

[89] X. Guo , B. Su , Z. Zhou , and J. Sha , “Rapid Evolution of Mammalian X‐Linked Testis microRNAs,” BMC Genomics 10 (2009): 97. 19257908 10.1186/1471-2164-10-97 PMC2660371

[90] L. J. Walport , R. J. Hopkinson , M. Vollmar , et al., “Human UTY(KDM6C) is a Male‐Specific Nϵ‐Methyl Lysyl Demethylase,” Journal of Biological Chemistry 289 (2014): 18302–18313. 24798337 10.1074/jbc.M114.555052 PMC4140284

[91] L. Yang , H. Li , Y. Zhao , et al., “Lysine Demethylases 6 A and 6B as Epigenetic Regulators in Therapeutic Resistance of Cancer,” Clinical Epigenetics 17 (2025): 198. 41272745 10.1186/s13148-025-02010-y PMC12639956

[92] M. G. Lee , J. Norman , A. Shilatifard , and R. Shiekhattar , “Physical and Functional Association of a Trimethyl H3K4 Demethylase and Ring6a/MBLR, a Polycomb‐Like Protein,” Cell 128 (2007): 877–887. 17320162 10.1016/j.cell.2007.02.004

[93] K. Horitani , N. W. Chavkin , Y. Arai , et al., “Disruption of the Uty Epigenetic Regulator Locus in Hematopoietic Cells Phenocopies the Profibrotic Attributes of Y Chromosome Loss in Heart Failure,” Nature Cardiovascular Research 3 (2024): 343–355. 10.1038/s44161-024-00441-z PMC11343478 39183958

[94] D. P. Czech , J. Lee , H. Sim , C. L. Parish , E. Vilain , and V. R. Harley , “The Human Testis‐Determining Factor SRY Localizes in Midbrain Dopamine Neurons and Regulates Multiple Components of Catecholamine Synthesis and Metabolism,” Journal of Neurochemistry 122 (2012): 260–271. 22568433 10.1111/j.1471-4159.2012.07782.x PMC3529967

[95] M. E. Turner , D. Ely , J. Prokop , and A. Milsted , “Sry, More Than Testis Determination?,” American Journal of Physiology. Regulatory, Integrative and Comparative Physiology 301 (2011): R561–R571. 21677270 10.1152/ajpregu.00645.2010

[96] A. Milsted , L. Serova , E. L. Sabban , G. Dunphy , M. E. Turner , and D. L. Ely , “Regulation of Tyrosine Hydroxylase Gene Transcription by Sry,” Neuroscience Letters 369 (2004): 203–207. 15464265 10.1016/j.neulet.2004.07.052

[97] A. Scanzano and M. Cosentino , “Adrenergic Regulation of Innate Immunity: A Review,” Frontiers in Pharmacology 6 (2015): 171. 26321956 10.3389/fphar.2015.00171 PMC4534859

[98] M. A. Flierl , D. Rittirsch , M. Huber‐Lang , J. V. Sarma , and P. A. Ward , “Catecholamines‐Crafty Weapons in the Inflammatory Arsenal of Immune/Inflammatory Cells or Opening Pandora's Box?,” Molecular Medicine 14 (2008): 195–204. 18079995 10.2119/2007-00105.Flierl PMC2136428

[99] O. O. Seminog , A. B. Seminog , D. Yeates , and M. J. Goldacre , “Associations Between Klinefelter's Syndrome and Autoimmune Diseases: English National Record Linkage Studies,” Autoimmunity 48 (2015): 125–128. 25295757 10.3109/08916934.2014.968918

[100] R. H. Scofield , G. R. Bruner , B. Namjou , et al., “Klinefelter's Syndrome (47,XXY) in Male Systemic Lupus Erythematosus Patients: Support for the Notion of a Gene‐Dose Effect From the X Chromosome,” Arthritis and Rheumatism 58 (2008): 2511–2517. 18668569 10.1002/art.23701 PMC2824898

[101] K. Liu , B. T. Kurien , S. L. Zimmerman , et al., “X Chromosome Dose and Sex Bias in Autoimmune Diseases: Increased Prevalence of 47,XXX in Systemic Lupus Erythematosus and Sjögren's Syndrome,” Arthritis & Rheumatology (Hoboken, N.J.) 68 (2016): 1290–1300. 26713507 10.1002/art.39560 PMC5019501

[102] N. Lefèvre , F. Corazza , J. Valsamis , et al., “The Number of X Chromosomes Influences Inflammatory Cytokine Production Following Toll‐Like Receptor Stimulation,” Frontiers in Immunology 10 (2019): 1052. 31143188 10.3389/fimmu.2019.01052 PMC6521177

[103] T. Kawai and S. Akira , “Signaling to NF‐kappaB by Toll‐Like Receptors,” Trends in Molecular Medicine 13 (2007): 460–469. 18029230 10.1016/j.molmed.2007.09.002

[104] J. Jantsch , M. Wiese , J. Schödel , et al., “Toll‐Like Receptor Activation and Hypoxia Use Distinct Signaling Pathways to Stabilize Hypoxia‐Inducible Factor 1α (HIF1A) and Result in Differential HIF1A‐Dependent Gene Expression,” Journal of Leukocyte Biology 90 (2011): 551–562. 21685248 10.1189/jlb.1210683

[105] H. Yao , K. Coppola , J. E. Schweig , F. Crawford , M. Mullan , and D. Paris , “Distinct Signaling Pathways Regulate TREM2 Phagocytic and NFκB Antagonistic Activities,” Frontiers in Cellular Neuroscience 13 (2019): 457. 31649511 10.3389/fncel.2019.00457 PMC6795686

[106] Z. Zhu , X. Zhang , W. Dong , et al., “TREM2 Suppresses the Proinflammatory Response to Facilitate PRRSV Infection via PI3K/NF‐κB Signaling,” PLoS Pathogens 16 (2020): e1008543. 32401783 10.1371/journal.ppat.1008543 PMC7250469

[107] M. T. R. Gomes , E. S. Guimarães , F. V. Marinho , et al., “STING Regulates Metabolic Reprogramming in Macrophages via HIF‐1α During Brucella Infection,” PLoS Pathogens 17 (2021): e1009597. 33989349 10.1371/journal.ppat.1009597 PMC8153530

[108] X. Hua , M. Bao , H. Mo , et al., “STING Regulates the Transformation of the Proinflammatory Macrophage Phenotype by HIF1A Into Autoimmune Myocarditis,” International Immunopharmacology 121 (2023): 110523. 37354779 10.1016/j.intimp.2023.110523

[109] E. Farahani , L. S. Reinert , R. Narita , et al., “The HIF Transcription Network Exerts Innate Antiviral Activity in Neurons and Limits Brain Inflammation,” Cell Reports 43 (2024): 113792. 38363679 10.1016/j.celrep.2024.113792 PMC10915869

[110] Y. Yan , S. Bai , H. Han , et al., “Knockdown of trem2 Promotes Proinflammatory Microglia and Inhibits Glioma Progression via the JAK2/STAT3 and NF‐κB Pathways,” Cell Communication and Signaling 22 (2024): 272. 38750472 10.1186/s12964-024-01642-6 PMC11094905

[111] M. Lin , J.‐X. Yu , W.‐X. Zhang , F.‐X. Lao , and H.‐C. Huang , “Roles of TREM2 in the Pathological Mechanism and the Therapeutic Strategies of Alzheimer's Disease,” Journal of Prevention of Alzheimer's Disease 11 (2024): 1682–1695. 10.14283/jpad.2024.164 PMC11573818 39559879

[112] D. Capece , D. Verzella , I. Flati , P. Arboretto , J. Cornice , and G. Franzoso , “NF‐κB: Blending Metabolism, Immunity, and Inflammation,” Trends in Immunology 43 (2022): 757–775. 35965153 10.1016/j.it.2022.07.004

[113] A. F. McGettrick and L. A. J. O'Neill , “The Role of HIF in Immunity and Inflammation,” Cell Metabolism 32 (2020): 524–536. 32853548 10.1016/j.cmet.2020.08.002

[114] S. E. Corcoran and L. A. J. O'Neill , “HIF1α and Metabolic Reprogramming in Inflammation,” Journal of Clinical Investigation 126 (2016): 3699–3707. 27571407 10.1172/JCI84431 PMC5096812

[115] L. A. J. O'Neill and E. J. Pearce , “Immunometabolism Governs Dendritic Cell and Macrophage Function,” Journal of Experimental Medicine 213 (2016): 15–23. 26694970 10.1084/jem.20151570 PMC4710204

[116] B. Kelly and L. A. J. O'Neill , “Metabolic Reprogramming in Macrophages and Dendritic Cells in Innate Immunity,” Cell Research 25 (2015): 771–784. 26045163 10.1038/cr.2015.68 PMC4493277

[117] S. Mishra , G. Bassi , and Y. X. Z. Xu , “Sex Differences in Immunometabolism: An Unexplored Area,” Methods in Molecular Biology 2184 (2020): 265–271. 32808231 10.1007/978-1-0716-0802-9_18

[118] M. Pujantell and M. Altfeld , “Consequences of Sex Differences in Type I IFN Responses for the Regulation of Antiviral Immunity,” Frontiers in Immunology 13 (2022): 986840. 36189206 10.3389/fimmu.2022.986840 PMC9522975

[119] Y. Huang , P. Yong , D. Dickey , S. M. Vora , H. Wu , and D. A. Bernlohr , “Inflammasome Activation and Pyroptosis via a Lipid‐Regulated SIRT1‐p53‐ASC Axis in Macrophages From Male Mice and Humans,” Endocrinology 163 (2022): bqac014. 35136993 10.1210/endocr/bqac014 PMC8896164

[120] M. E. Raichle and D. A. Gusnard , “Appraising the Brain's Energy Budget,” Proceedings of the National Academy of Sciences of the United States of America 99 (2002): 10237–10239. 12149485 10.1073/pnas.172399499 PMC124895

[121] M. K. Jha and B. M. Morrison , “Glia‐Neuron Energy Metabolism in Health and Diseases: New Insights Into the Role of Nervous System Metabolic Transporters,” Experimental Neurology 309 (2018): 23–31. 30044944 10.1016/j.expneurol.2018.07.009 PMC6156776

[122] E. S. Jung , H. Choi , and I. Mook‐Jung , “Decoding Microglial Immunometabolism: A New Frontier in Alzheimer's Disease Research,” Molecular Neurodegeneration 20 (2025): 37. 40149001 10.1186/s13024-025-00825-0 PMC11948825

[123] M. Huang , A. Long , L. Hao , Z. Shi , and M. Zhang , “Astrocyte in Neurological Disease: Pathogenesis and Therapy,” MedComm 6 (2025): e70299. 40686921 10.1002/mco2.70299 PMC12271643

[124] A. A. Nugent , K. Lin , B. van Lengerich , et al., “TREM2 Regulates Microglial Cholesterol Metabolism Upon Chronic Phagocytic Challenge,” Neuron 105 (2020): 837–854.e9. 31902528 10.1016/j.neuron.2019.12.007

[125] Y. Wang , M. Cella , K. Mallinson , et al., “TREM2 Lipid Sensing Sustains the Microglial Response in an Alzheimer's Disease Model,” Cell 160 (2015): 1061–1071. 25728668 10.1016/j.cell.2015.01.049 PMC4477963

[126] T. K. Ulland , W. M. Song , S. C. C. Huang , et al., “TREM2 Maintains Microglial Metabolic Fitness in Alzheimer's Disease,” Cell 170 (2017): 649–663.e13. 28802038 10.1016/j.cell.2017.07.023 PMC5573224

[127] B. Dos Santos , T. Piermartiri , and C. I. Tasca , “The Impact of Purine Nucleosides on Neuroplasticity in the Adult Brain,” Purinergic Signal 21 (2025): 113–131. 38367178 10.1007/s11302-024-09988-9 PMC11958884

[128] M. Tewari , S. Michalski , and T. M. Egan , “Modulation of Microglial Function by ATP‐Gated P2X7 Receptors: Studies in Rat, Mice and Human,” Cells 13 (2024): 161. 38247852 10.3390/cells13020161 PMC10814008

[129] Y. Zhang , P. Jia , K. Wang , et al., “Lactate Modulates Microglial Inflammatory Responses After Oxygen‐Glucose Deprivation Through HIF‐1α‐Mediated Inhibition of NF‐κB,” Brain Research Bulletin 195 (2023): 1–13. 36746287 10.1016/j.brainresbull.2023.02.002

[130] S.‐J. Liu , Y. Zhong , X. Y. You , W. H. Liu , A. Q. Li , and S. M. Liu , “Insulin‐Like Growth Factor 1 Opposes the Effects of C‐Reactive Protein on Endothelial Cell Activation,” Molecular and Cellular Biochemistry 385 (2014): 199–205. 24065393 10.1007/s11010-013-1828-y

[131] D. Laketa and I. Lavrnja , “Extracellular Purine Metabolism‐Potential Target in Multiple Sclerosis,” Molecular Neurobiology 61 (2024): 8361–8386. 38499905 10.1007/s12035-024-04104-9

[132] B. Sperlágh and P. Illes , “Purinergic Modulation of Microglial Cell Activation,” Purinergic Signal 3 (2007): 117–127. 18404425 10.1007/s11302-006-9043-x PMC2096753

[133] B. F. Osborne , A. Turano , and J. M. Schwarz , “Sex Differences in the Neuroimmune System,” Current Opinion in Behavioral Sciences 23 (2018): 118–123. 30014014 10.1016/j.cobeha.2018.05.007 PMC6044467

[134] T. Hu , C.‐H. Liu , M. Lei , et al., “Metabolic Regulation of the Immune System in Health and Diseases: Mechanisms and Interventions,” Signal Transduction and Targeted Therapy 9 (2024): 268. 39379377 10.1038/s41392-024-01954-6 PMC11461632

[135] Z. Hu , X. Yu , R. Ding , et al., “Glycolysis Drives STING Signaling to Facilitate Dendritic Cell Antitumor Function,” Journal of Clinical Investigation 133 (2023): e166031. 36821379 10.1172/JCI166031 PMC10065062

[136] A. Viola , F. Munari , R. Sánchez‐Rodríguez , T. Scolaro , and A. Castegna , “The Metabolic Signature of Macrophage Responses,” Frontiers in Immunology 10 (2019): 1462. 31333642 10.3389/fimmu.2019.01462 PMC6618143

[137] L. A. J. O'Neill , R. J. Kishton , and J. Rathmell , “A Guide to Immunometabolism for Immunologists,” Nature Reviews Immunology 16 (2016): 553–565. 10.1038/nri.2016.70 PMC5001910 27396447

[138] H. Yu , Q. Chang , T. Sun , et al., “Metabolic Reprogramming and Polarization of Microglia in Parkinson's Disease: Role of Inflammasome and Iron,” Ageing Research Reviews 90 (2023): 102032. 37572760 10.1016/j.arr.2023.102032

[139] J. Lu , C. Wang , X. Cheng , et al., “A Breakdown in Microglial Metabolic Reprogramming Causes Internalization Dysfunction of α‐Synuclein in a Mouse Model of Parkinson's Disease,” Journal of Neuroinflammation 19 (2022): 113. 35599331 10.1186/s12974-022-02484-0 PMC9124408

[140] J. Miao , L. Chen , X. Pan , L. Li , B. Zhao , and J. Lan , “Microglial Metabolic Reprogramming: Emerging Insights and Therapeutic Strategies in Neurodegenerative Diseases,” Cellular and Molecular Neurobiology 43 (2023): 3191–3210. 37341833 10.1007/s10571-023-01376-y PMC11410021

[141] X. Li , C. Fang , Y. Li , X. Xiong , X. Xu , and L. Gu , “Glycolytic Reprogramming During Microglial Polarization in Neurological Diseases,” Frontiers in Immunology 16 (2025): 1648887. 41126831 10.3389/fimmu.2025.1648887 PMC12537410

[142] N. Lu , Z. Jin , N. Liu , C. Zhu , H. Wei , and Q. Xu , “Microglial Glycolytic Reprogramming in Alzheimer's Disease: Association With Impaired Phagocytic Function and Altered Vascular Proximity,” Journal of Neuroinflammation 22 (2025): 223. 41039597 10.1186/s12974-025-03546-9 PMC12492836

[143] X. Zhang , N. Alshakhshir , and L. Zhao , “Glycolytic Metabolism, Brain Resilience, and Alzheimer's Disease,” Frontiers in Neuroscience 15 (2021): 662242. 33994936 10.3389/fnins.2021.662242 PMC8113697

[144] B. E. Clarke and R. Patani , “The Microglial Component of Amyotrophic Lateral Sclerosis,” Brain 143 (2020): 3526–3539. 33427296 10.1093/brain/awaa309 PMC7805793

[145] T. W. Tefera , F. J. Steyn , S. T. Ngo , and K. Borges , “CNS Glucose Metabolism in Amyotrophic Lateral Sclerosis: A Therapeutic Target?,” Cell & Bioscience 11 (2021): 14. 33431046 10.1186/s13578-020-00511-2 PMC7798275

[146] J. Cheng , R. Zhang , Z. Xu , et al., “Early Glycolytic Reprogramming Controls Microglial Inflammatory Activation,” Journal of Neuroinflammation 18 (2021): 129. 34107997 10.1186/s12974-021-02187-y PMC8191212

[147] L. Wang , S. Pavlou , X. Du , M. Bhuckory , H. Xu , and M. Chen , “Glucose Transporter 1 Critically Controls Microglial Activation Through Facilitating Glycolysis,” Molecular Neurodegeneration 14 (2019): 2. 30634998 10.1186/s13024-019-0305-9 PMC6329071

[148] Y. Li , W. Long , M. Gao , et al., “TREM2 Regulates High Glucose‐Induced Microglial Inflammation via the NLRP3 Signaling Pathway,” Brain Sciences 11 (2021): 896. 34356130 10.3390/brainsci11070896 PMC8306970

[149] G. M. Tannahill , A. M. Curtis , J. Adamik , et al., “Succinate Is an Inflammatory Signal That Induces IL‐1β Through HIF‐1α,” Nature 496 (2013): 238–242. 23535595 10.1038/nature11986 PMC4031686

[150] E. L. Mills , B. Kelly , A. Logan , et al., “Succinate Dehydrogenase Supports Metabolic Repurposing of Mitochondria to Drive Inflammatory Macrophages,” Cell 167 (2016): 457–470.e13. 27667687 10.1016/j.cell.2016.08.064 PMC5863951

[151] K. Monsorno , A. Buckinx , and R. C. Paolicelli , “Microglial Metabolic Flexibility: Emerging Roles for Lactate,” Trends in Endocrinology and Metabolism 33 (2022): 186–195. 34996673 10.1016/j.tem.2021.12.001

[152] C. Yang , R.‐Y. Pan , F. Guan , and Z. Yuan , “Lactate Metabolism in Neurodegenerative Diseases,” Neural Regeneration Research 19 (2024): 69–74. 37488846 10.4103/1673-5374.374142 PMC10479854

[153] Z. Li , W. Zheng , W. Kong , and T. Zeng , “Itaconate: A Potent Macrophage Immunomodulator,” Inflammation 46 (2023): 1177–1191. 37142886 10.1007/s10753-023-01819-0 PMC10159227

[154] W. Yang , Y. Wang , K. Tao , and R. Li , “Metabolite Itaconate in Host Immunoregulation and Defense,” Cellular and Molecular Biology Letters 28 (2023): 100. 38042791 10.1186/s11658-023-00503-3 PMC10693715

[155] J. Escrivà‐Font , T. Cao , and C. R. Consiglio , “Decoding Sex Differences in Human Immunity Through Systems Immunology,” Oxford Open Immunology 6 (2025): iqaf006. 40692743 10.1093/oxfimm/iqaf006 PMC12279299

[156] J. J. Chang , M. Woods , R. J. Lindsay , et al., “Higher Expression of Several Interferon‐Stimulated Genes in HIV‐1‐Infected Females After Adjusting for the Level of Viral Replication,” Journal of Infectious Diseases 208 (2013): 830–838. 23757341 10.1093/infdis/jit262 PMC3733517

[157] N. Sauerwald , Z. Zhang , I. Ramos , et al., “Pre‐Infection Antiviral Innate Immunity Contributes to Sex Differences in SARS‐CoV‐2 Infection,” Cell Systems 13 (2022): 924–931.e4. 36323307 10.1016/j.cels.2022.10.005 PMC9623453

[158] S. Pace and O. Werz , “Impact of Androgens on Inflammation‐Related Lipid Mediator Biosynthesis in Innate Immune Cells,” Frontiers in Immunology 11 (2020): 1356. 32714332 10.3389/fimmu.2020.01356 PMC7344291

[159] A. C. Kwan , M. Wang , H. Ji , et al., “Sex‐Divergent Blood Pressure Associations With Multiorgan System Metabolic Stress‐Brief Report,” Arteriosclerosis, Thrombosis, and Vascular Biology 45 (2025): 557–561. 40013361 10.1161/ATVBAHA.124.322169 PMC11936467

[160] M.‐V. Guillot‐Sestier , A. R. Araiz , V. Mela , et al., “Microglial Metabolism Is a Pivotal Factor in Sexual Dimorphism in Alzheimer's Disease,” Communications Biology 4 (2021): 711. 34112929 10.1038/s42003-021-02259-y PMC8192523

[161] M. A. Lynch , “Exploring Sex‐Related Differences in Microglia May Be a Game‐Changer in Precision Medicine,” Frontiers in Aging Neuroscience 14 (2022): 868448. 35431903 10.3389/fnagi.2022.868448 PMC9009390

[162] J. Han , Y. Fan , K. Zhou , K. Blomgren , and R. A. Harris , “Uncovering Sex Differences of Rodent Microglia,” Journal of Neuroinflammation 18 (2021): 74. 33731174 10.1186/s12974-021-02124-z PMC7972194

[163] Y. Hou , J. Z. K. Caldwell , J. D. Lathia , et al., “Microglial Immunometabolism Endophenotypes Contribute to Sex Difference in Alzheimer's Disease,” Alzheimer's & Dementia 20 (2024): 1334–1349. 10.1002/alz.13546 PMC10916937 37985399

[164] E. Di Martino , A. Ambikan , D. Ramsköld , et al., “Inflammatory, Metabolic, and Sex‐Dependent Gene‐Regulatory Dynamics of Microglia and Macrophages in Neonatal Hippocampus After Hypoxia‐Ischemia,” IScience 27 (2024): 109346. 38500830 10.1016/j.isci.2024.109346 PMC10945260

[165] B. Chausse , P. A. Kakimoto , and O. Kann , “Microglia and Lipids: How Metabolism Controls Brain Innate Immunity,” Seminars in Cell & Developmental Biology 112 (2021): 137–144. 32807643 10.1016/j.semcdb.2020.08.001

[166] E. C. Damisah , A. Rai , and J. Grutzendler , “TREM2: Modulator of Lipid Metabolism in Microglia,” Neuron 105 (2020): 759–761. 32135085 10.1016/j.neuron.2020.02.008

[167] K. G. Sprenger , E. E. Lietzke , J. T. Melchior , and K. D. Bruce , “Lipid and Lipoprotein Metabolism in Microglia: Alzheimer's Disease Mechanisms and Interventions,” Journal of Lipid Research 66 (2025): 100872. 40769380 10.1016/j.jlr.2025.100872 PMC12538436

[168] C. de Dios , X. Abadin , V. Roca‐Agujetas , et al., “Inflammasome Activation Under High Cholesterol Load Triggers a Protective Microglial Phenotype While Promoting Neuronal Pyroptosis,” Translational Neurodegeneration 12 (2023): 10. 36895045 10.1186/s40035-023-00343-3 PMC9996936

[169] Z. Xu , S. Kiani Shabestari , S. Barannikov , et al., “Microglia‐Specific Regulation of Lipid Metabolism in Alzheimer's Disease Revealed by Microglial Depletion in 5xFAD Mice,” Nature Communications 16 (2025): 9156. 10.1038/s41467-025-64161-z PMC12528747 41093842

[170] P. J. Paasila , J. A. Aramideh , G. T. Sutherland , and M. B. Graeber , “Synapses, Microglia, and Lipids in Alzheimer's Disease,” Frontiers in Neuroscience 15 (2021): 778822. 35095394 10.3389/fnins.2021.778822 PMC8789683

[171] P. Duewell , H. Kono , K. J. Rayner , et al., “NLRP3 Inflammasomes Are Required for Atherogenesis and Activated by Cholesterol Crystals,” Nature 464 (2010): 1357–1361. 20428172 10.1038/nature08938 PMC2946640

[172] R. Zhou , A. Tardivel , B. Thorens , I. Choi , and J. Tschopp , “Thioredoxin‐Interacting Protein Links Oxidative Stress to Inflammasome Activation,” Nature Immunology 11 (2010): 136–140. 20023662 10.1038/ni.1831

[173] E. A. Dennis and P. C. Norris , “Eicosanoid Storm in Infection and Inflammation,” Nature Reviews. Immunology 15 (2015): 511–523. 10.1038/nri3859 PMC4606863 26139350

[174] Y.‐H. Han , K. Lee , A. Saha , et al., “Specialized Proresolving Mediators for Therapeutic Interventions Targeting Metabolic and Inflammatory Disorders,” Biomolecules & Therapeutics 29 (2021): 455–464. 34162770 10.4062/biomolther.2021.094 PMC8411019

[175] V. Dela Justina , F. R. Giachini , J. C. Sullivan , and R. C. Webb , “Toll‐Like Receptors Contribute to Sex Differences in Blood Pressure Regulation,” Journal of Cardiovascular Pharmacology 76 (2020): 255–266. 32902942 10.1097/FJC.0000000000000869 PMC7751064

[176] A. Popotas , G. J. Casimir , F. Corazza , and N. Lefèvre , “Sex‐Related Immunity: Could Toll‐Like Receptors Be the Answer in Acute Inflammatory Response?,” Frontiers in Immunology 15 (2024): 1379754. 38835761 10.3389/fimmu.2024.1379754 PMC11148260

[177] R. M. Craft , J. A. Marusich , and J. L. Wiley , “Sex Differences in Cannabinoid Pharmacology: A Reflection of Differences in the Endocannabinoid System?,” Life Sciences 92 (2013): 476–481. 22728714 10.1016/j.lfs.2012.06.009 PMC3492530

[178] J. Giorgio , C. Jonson , Y. Wang , J. S. Yokoyama , J. Wang , and W. J. Jagust , “Variable and Interactive Effects of Sex, APOE ε4 and TREM2 on the Deposition of Tau in Entorhinal and Neocortical Regions,” Nature Communications 16 (2025): 5812. 10.1038/s41467-025-60370-8 PMC12214702 40595476

[179] D. Wu , X. Bi , and K. H.‐M. Chow , “Identification of Female‐Enriched and Disease‐Associated Microglia (FDAMic) Contributes to Sexual Dimorphism in Late‐Onset Alzheimer's Disease,” Journal of Neuroinflammation 21 (2024): 1. 38178204 10.1186/s12974-023-02987-4 PMC10765928

[180] S. H. Gerges and A. O. S. El‐Kadi , “Sex Differences in Eicosanoid Formation and Metabolism: A Possible Mediator of Sex Discrepancies in Cardiovascular Diseases,” Pharmacology & Therapeutics 234 (2022): 108046. 34808133 10.1016/j.pharmthera.2021.108046

[181] J. So , J. H. Yao , R. Magadmi , N. R. Matthan , and S. Lamon‐Fava , “Sex Differences in Lipid Mediators Derived From Omega‐3 Fatty Acids in Older Individuals With Low‐Grade Chronic Inflammation,” Prostaglandins, Leukotrienes, and Essential Fatty Acids 203 (2024): 102655. 39488904 10.1016/j.plefa.2024.102655 PMC11624983

[182] Y. Kaljas , C. Liu , M. Skaldin , et al., “Human Adenosine Deaminases ADA1 and ADA2 Bind to Different Subsets of Immune Cells,” Cellular and Molecular Life Sciences 74 (2017): 555–570. 27663683 10.1007/s00018-016-2357-0 PMC11107696

[183] Z.‐W. Gao , Z. Gao , L. Yang , et al., “Distinct Roles of Adenosine Deaminase Isoenzymes ADA1 and ADA2: A Pan‐Cancer Analysis,” Frontiers in Immunology 13 (2022): 903461. 35663977 10.3389/fimmu.2022.903461 PMC9157497

[184] S. Signa , A. Bertoni , F. Penco , et al., “Adenosine Deaminase 2 Deficiency (DADA2): A Crosstalk Between Innate and Adaptive Immunity,” Frontiers in Immunology 13 (2022): 935957. 35898506 10.3389/fimmu.2022.935957 PMC9309328

[185] P. Illes , P. Rubini , H. Ulrich , Y. Zhao , and Y. Tang , “Regulation of Microglial Functions by Purinergic Mechanisms in the Healthy and Diseased CNS,” Cells 9 (2020): 1108. 32365642 10.3390/cells9051108 PMC7290360

[186] K. E. Campagno , W. Lu , A. H. Jassim , et al., “Rapid Morphologic Changes to Microglial Cells and Upregulation of Mixed Microglial Activation State Markers Induced by P2X7 Receptor Stimulation and Increased Intraocular Pressure,” Journal of Neuroinflammation 18 (2021): 217. 34544431 10.1186/s12974-021-02251-7 PMC8454080

[187] H. Zarrinmayeh and P. R. Territo , “Purinergic Receptors of the Central Nervous System: Biology, PET Ligands, and Their Applications,” Molecular Imaging 19 (2020): 1536012120927609. 32539522 10.1177/1536012120927609 PMC7297484

[188] G. Haskó , J. Linden , B. Cronstein , and P. Pacher , “Adenosine Receptors: Therapeutic Aspects for Inflammatory and Immune Diseases,” Nature Reviews. Drug Discovery 7 (2008): 759–770. 18758473 10.1038/nrd2638 PMC2568887

[189] G. Pallio and F. Mannino , “Non‐Canonical Functions of Adenosine Receptors: Emerging Roles in Metabolism, Immunometabolism, and Epigenetic Regulation,” International Journal of Molecular Sciences 26 (2025): 7241. 40806376 10.3390/ijms26157241 PMC12346349

[190] K. Köröskényi , B. Kiss , and Z. Szondy , “Adenosine A2A Receptor Signaling Attenuates LPS‐Induced Pro‐Inflammatory Cytokine Formation of Mouse Macrophages by Inducing the Expression of DUSP1,” Biochimica et Biophysica Acta 1863 (2016): 1461–1471. 27066978 10.1016/j.bbamcr.2016.04.003

[191] J. G. McLarnon , “Purinergic Mediated Changes in Ca2+ Mobilization and Functional Responses in Microglia: Effects of Low Levels of ATP,” Journal of Neuroscience Research 81 (2005): 349–356. 15948175 10.1002/jnr.20475

[192] B. Readhead , J. V. Haure‐Mirande , C. C. Funk , et al., “Multiscale Analysis of Independent Alzheimer's Cohorts Finds Disruption of Molecular, Genetic, and Clinical Networks by Human Herpesvirus,” Neuron 99 (2018): 64–82.e7. 29937276 10.1016/j.neuron.2018.05.023 PMC6551233

[193] P. Dembny , A. G. Newman , M. Singh , et al., “Human Endogenous Retrovirus HERV‐K(HML‐2) RNA Causes Neurodegeneration Through Toll‐Like Receptors,” JCI Insight 5 (2020): e131093. 32271161 10.1172/jci.insight.131093 PMC7205273

[194] G. Morris , M. Maes , M. Murdjeva , and B. K. Puri , “Do Human Endogenous Retroviruses Contribute to Multiple Sclerosis, and if So, How?,” Molecular Neurobiology 56 (2019): 2590–2605. 30047100 10.1007/s12035-018-1255-x PMC6459794

[195] B. Charvet , J. Pierquin , J. Brunel , et al., “Human Endogenous Retrovirus Type W Envelope From Multiple Sclerosis Demyelinating Lesions Shows Unique Solubility and Antigenic Characteristics,” Virologica Sinica 36 (2021): 1006–1026. 33770381 10.1007/s12250-021-00372-0 PMC8558138

[196] E. R. Roy , B. Wang , Y. W. Wan , et al., “Type I Interferon Response Drives Neuroinflammation and Synapse Loss in Alzheimer Disease,” Journal of Clinical Investigation 130 (2020): 1912–1930. 31917687 10.1172/JCI133737 PMC7108898

[197] E. Roy and W. Cao , “Glial Interference: Impact of Type I Interferon in Neurodegenerative Diseases,” Molecular Neurodegeneration 17 (2022): 78. 36435817 10.1186/s13024-022-00583-3 PMC9701358

[198] Y. Yoshida , Z. Chen , R. L. Baudier , et al., “Sex Differences in the Progression of Metabolic Risk Factors in Diabetes Development,” JAMA Network Open 5 (2022): e2222070. 35834256 10.1001/jamanetworkopen.2022.22070 PMC9284329

[199] M. Matos‐Silva , F. S. Lira , and B. M. Antunes , “Immunometabolic Insights Into Women's Health Across All Ages,” Maturitas 202 (2025): 108719. 40961729 10.1016/j.maturitas.2025.108719

[200] S. Kang , E. Y. Ko , A. E. Andrews , et al., “Microglia Undergo Sex‐Dimorphic Transcriptional and Metabolic Rewiring During Aging,” Journal of Neuroinflammation 21 (2024): 150. 38840206 10.1186/s12974-024-03130-7 PMC11155174

[201] N. Alvarez‐Sanchez and S. E. Dunn , “Potential Biological Contributers to the Sex Difference in Multiple Sclerosis Progression,” Frontiers in Immunology 14 (2023): 1175874. 37122747 10.3389/fimmu.2023.1175874 PMC10140530

[202] B. L. Guerrero and N. L. Sicotte , “Microglia in Multiple Sclerosis: Friend or Foe?,” Frontiers in Immunology 11 (2020): 374. 32265902 10.3389/fimmu.2020.00374 PMC7098953

[203] M. G. Tansey , R. L. Wallings , M. C. Houser , M. K. Herrick , C. E. Keating , and V. Joers , “Inflammation and Immune Dysfunction in Parkinson Disease,” Nature Reviews Immunology 22 (2022): 657–673. 10.1038/s41577-022-00684-6 PMC8895080 35246670

[204] M. Bourque , M. Morissette , D. Soulet , and T. Di Paolo , “Impact of Sex on Neuroimmune Contributions to Parkinson's Disease,” Brain Research Bulletin 199 (2023): 110668. 37196734 10.1016/j.brainresbull.2023.110668

[205] L. Caldi Gomes , S. Hänzelmann , F. Hausmann , et al., “Multiomic ALS Signatures Highlight Subclusters and Sex Differences Suggesting the MAPK Pathway as Therapeutic Target,” Nature Communications 15 (2024): 4893. 10.1038/s41467-024-49196-y PMC11161513 38849340

[206] L. E. Korshoj and T. Kielian , “Neuroimmune Metabolism: Uncovering the Role of Metabolic Reprogramming in Central Nervous System Disease,” Journal of Neurochemistry 158 (2021): 8–13. 33993505 10.1111/jnc.15376

[207] F. Di Virgilio and M. Vuerich , “Purinergic Signaling in the Immune System,” Autonomic Neuroscience 191 (2015): 117–123. 25979766 10.1016/j.autneu.2015.04.011

[208] T. C. Browne , K. McQuillan , R. M. McManus , J.‐A. O'Reilly , K. H. G. Mills , and M. A. Lynch , “IFN‐γ Production by Amyloid β‐Specific Th1 Cells Promotes Microglial Activation and Increases Plaque Burden in a Mouse Model of Alzheimer's Disease,” Journal of Immunology (Baltimore, Md.: 1950) 190 (2013): 2241–2251. 23365075 10.4049/jimmunol.1200947

[209] M. A. de Dias Sousa , C. S. Desidério , J. da Silva Catarino , et al., “Role of Cytokines, Chemokines and IFN‐γ+ IL‐17+ Double‐Positive CD4+ T Cells in Patients With Multiple Sclerosis,” Biomedicine 10 (2022): 2062. 10.3390/biomedicines10092062 PMC9495686 36140164

[210] A. Litwiniuk , A. Baranowska‐Bik , A. Domańska , M. Kalisz , and W. Bik , “Contribution of Mitochondrial Dysfunction Combined With NLRP3 Inflammasome Activation in Selected Neurodegenerative Diseases,” Pharmaceuticals (Basel, Switzerland) 14 (2021): 1221. 34959622 10.3390/ph14121221 PMC8703835

[211] A. Kikuchi , A. Takeda , H. Onodera , et al., “Systemic Increase of Oxidative Nucleic Acid Damage in Parkinson's Disease and Multiple System Atrophy,” Neurobiology of Disease 9 (2002): 244–248. 11895375 10.1006/nbdi.2002.0466

[212] J. Rainger , M. Keighren , D. R. Keene , et al., “A Trans‐Acting Protein Effect Causes Severe Eye Malformation in the Mp Mouse,” PLoS Genetics 9 (2013): e1003998. 24348270 10.1371/journal.pgen.1003998 PMC3861116

[213] T. T. B. Vo , T. T. Vo , E. M. Jung , et al., “Di‐(2 Ethylhexyl) Phthalate and Flutamide Alter Gene Expression in the Testis of Immature Male Rats,” Reproductive Biology and Endocrinology: RB&E 7 (2009): 104. 19781091 10.1186/1477-7827-7-104 PMC2760555

[214] O. H. Kantarci , A. Goris , D. D. Hebrink , et al., “IFNG Polymorphisms Are Associated With Gender Differences in Susceptibility to Multiple Sclerosis,” Genes and Immunity 6 (2005): 153–161. 15674394 10.1038/sj.gene.6364164

[215] S. Quan , X. Fu , H. Cai , Z. Ren , Y. Xu , and L. Jia , “The Neuroimmune Nexus: Unraveling the Role of the mtDNA‐cGAS‐STING Signal Pathway in Alzheimer's Disease,” Molecular Neurodegeneration 20 (2025): 25. 40038765 10.1186/s13024-025-00815-2 PMC11877805

[216] M. F. Gulen , N. Samson , A. Keller , et al., “cGAS‐STING Drives Ageing‐Related Inflammation and Neurodegeneration,” Nature 620 (2023): 374–380. 37532932 10.1038/s41586-023-06373-1 PMC10412454

[217] S. Chung , J. H. Jeong , J. C. Park , et al., “Blockade of STING Activation Alleviates Microglial Dysfunction and a Broad Spectrum of Alzheimer's Disease Pathologies,” Experimental & Molecular Medicine 56 (2024): 1936–1951. 39218977 10.1038/s12276-024-01295-y PMC11447230

[218] A. Deczkowska , O. Matcovitch‐Natan , A. Tsitsou‐Kampeli , et al., “Mef2C Restrains Microglial Inflammatory Response and Is Lost in Brain Ageing in an IFN‐I‐Dependent Manner,” Nature Communications 8 (2017): 717. 10.1038/s41467-017-00769-0 PMC5620041 28959042

[219] C. Marques , A. Held , K. Dorfman , et al., “Neuronal STING Activation in Amyotrophic Lateral Sclerosis and Frontotemporal Dementia,” Acta Neuropathologica 147 (2024): 56. 38478117 10.1007/s00401-024-02688-z PMC10937762

[220] Ú. Franco‐Enzástiga , N. N. Inturi , K. Natarajan , et al., “Epigenomic Landscape of the Human Dorsal Root Ganglion: Sex Differences and Transcriptional Regulation of Nociceptive Genes,” preprint, bioRxivorg (2024), 10.1101/2024.03.27.587047. PMC11819886 39928726

[221] M. M. El‐Mas and A. A. Abdel‐Rahman , “Estrogen Modulation of the Ethanol‐Evoked Myocardial Oxidative Stress and Dysfunction via DAPK3/Akt/ERK Activation in Male Rats,” Toxicology and Applied Pharmacology 287 (2015): 284–292. 26111663 10.1016/j.taap.2015.06.015 PMC4549171

[222] P. Leister , A. Felten , A. I. Chasan , and K. H. Scheidtmann , “ZIP Kinase Plays a Crucial Role in Androgen Receptor‐Mediated Transcription,” Oncogene 27 (2008): 3292–3300. 18084323 10.1038/sj.onc.1210995

[223] V. Vijay , T. Han , C. L. Moland , J. C. Kwekel , J. C. Fuscoe , and V. G. Desai , “Sexual Dimorphism in the Expression of Mitochondria‐Related Genes in Rat Heart at Different Ages,” PLoS One 10 (2015): e0117047. 25615628 10.1371/journal.pone.0117047 PMC4304718

[224] C. Cattaneo and J. Pagonabarraga , “Sex Differences in Parkinson's Disease: A Narrative Review,” Neurology and Therapy 14 (2025): 57–70. 39630386 10.1007/s40120-024-00687-6 PMC11762054

[225] T. G. Demarest and M. M. McCarthy , “Sex Differences in Mitochondrial (Dys)function: Implications for Neuroprotection,” Journal of Bioenergetics and Biomembranes 47 (2015): 173–188. 25293493 10.1007/s10863-014-9583-7 PMC4988325

[226] D. Cacabelos , O. Ramírez‐Núñez , A. B. Granado‐Serrano , et al., “Early and Gender‐Specific Differences in Spinal Cord Mitochondrial Function and Oxidative Stress Markers in a Mouse Model of ALS,” Acta Neuropathologica Communications 4 (2016): 3. 26757991 10.1186/s40478-015-0271-6 PMC4711180

[227] V. López‐López , G. Iniesta , M. Galán‐Ganga , et al., “Sex‐Dependent Interferon Signaling Drives Female‐Biased Vulnerability in Alzheimer's Disease,” preprint, bioRxiv (2025), 10.1101/2025.08.22.671724. PMC13312654 42083037

[228] G. K. Carling , L. Fan , N. R. Foxe , et al., “Alzheimer's Disease‐Linked Risk Alleles Elevate Microglial cGAS‐Associated Senescence and Neurodegeneration in a Tauopathy Model,” Neuron 112 (2024): 3877–3896.e8. 39353433 10.1016/j.neuron.2024.09.006 PMC11624100

[229] J. Guo , Y. Tang , and P. Illes , “The Enzymatic Degradation of ATP to Adenosine by Microglial CD39 Regulates Neurovascular Coupling and Metabolic Supply to the Brain,” Purinergic Signal 21 (2025): 979–981. 40540133 10.1007/s11302-025-10102-w PMC12454696

[230] B. Kutryb‐Zajac , A. Kawecka , F. Caratis , et al., “The Impaired Distribution of Adenosine Deaminase Isoenzymes in Multiple Sclerosis Plasma and Cerebrospinal Fluid,” Frontiers in Molecular Neuroscience 15 (2022): 998023. 36204140 10.3389/fnmol.2022.998023 PMC9530629

[231] I. T. Mathews , P. Saminathan , M. Henglin , et al., “Linoleoyl‐Lysophosphatidylcholine Suppresses Immune‐Related Adverse Events due to Immune Checkpoint Blockade,” preprint, medRxiv (2024), 10.1101/2024.08.07.24310974.

[232] Y. Dong , J. Lagarde , L. Xicota , et al., “Neutrophil Hyperactivation Correlates With Alzheimer's Disease Progression,” Annals of Neurology 83 (2018): 387–405. 29369398 10.1002/ana.25159

[233] X. Zha , X. Liu , M. Wei , et al., “Microbiota‐Derived Lysophosphatidylcholine Alleviates Alzheimer's Disease Pathology via Suppressing Ferroptosis,” Cell Metabolism 37 (2025): 169–186.e9. 39510074 10.1016/j.cmet.2024.10.006

[234] E. R. Bowman , M. Kulkarni , J. Gabriel , et al., “Altered Lipidome Composition Is Related to Markers of Monocyte and Immune Activation in Antiretroviral Therapy Treated Human Immunodeficiency Virus (HIV) Infection and in Uninfected Persons,” Frontiers in Immunology 10 (2019): 785. 31040846 10.3389/fimmu.2019.00785 PMC6477036

[235] V. Kalia , D. Reyes‐Dumeyer , S. Dubey , et al., “Lysophosphatidylcholines Are Associated With Amyloidosis in Early Stages of Alzheimer's Disease,” Nature Aging 6 (2025): 221–234, 10.1038/s43587-025-01025-7. 41407938 PMC12823400

[236] S.‐H. Law , M. L. Chan , G. K. Marathe , F. Parveen , C. H. Chen , and L. Y. Ke , “An Updated Review of Lysophosphatidylcholine Metabolism in Human Diseases,” International Journal of Molecular Sciences 20 (2019): 1149. 30845751 10.3390/ijms20051149 PMC6429061

[237] K. Eisinger , G. Liebisch , G. Schmitz , C. Aslanidis , S. Krautbauer , and C. Buechler , “Lipidomic Analysis of Serum From High Fat Diet Induced Obese Mice,” International Journal of Molecular Sciences 15 (2014): 2991–3002. 24562328 10.3390/ijms15022991 PMC3958895

[238] P. J. Ojala , T. E. Hirvonen , M. Hermansson , P. Somerharju , and J. Parkkinen , “Acyl Chain‐Dependent Effect of Lysophosphatidylcholine on Human Neutrophils,” Journal of Leukocyte Biology 82 (2007): 1501–1509. 17884992 10.1189/jlb.0507292

[239] R. D. Semba , “Perspective: The Potential Role of Circulating Lysophosphatidylcholine in Neuroprotection Against Alzheimer Disease,” Advances in Nutrition 11 (2020): 760–772. 32190891 10.1093/advances/nmaa024 PMC7360459

[240] V. Pavel , P. Mester , M. Höring , et al., “Distinct Plasma LPC Signatures Differentiate COVID‐19 Sepsis From Other Sepsis Aetiologies,” Biomedicine 13 (2025): 2110. 10.3390/biomedicines13092110 PMC12466981 41007672

[241] M. Gonzalez‐Freire , R. Moaddel , K. Sun , et al., “Targeted Metabolomics Shows Low Plasma Lysophosphatidylcholine 18:2 Predicts Greater Decline of Gait Speed in Older Adults: The Baltimore Longitudinal Study of Aging,” Journals of Gerontology. Series A, Biological Sciences and Medical Sciences 74 (2019): 62–67. 29788121 10.1093/gerona/gly100 PMC6298185

[242] P. Liu , W. Zhu , C. Chen , et al., “The Mechanisms of Lysophosphatidylcholine in the Development of Diseases,” Life Sciences 247 (2020): 117443. 32084434 10.1016/j.lfs.2020.117443

[243] Q. Zhou , Y. Chen , Y. Liang , and Y. Sun , “The Role of Lysophospholipid Metabolites LPC and LPA in the Pathogenesis of Chronic Obstructive Pulmonary Disease,” Metabolites 14 (2024): 317. 38921452 10.3390/metabo14060317 PMC11205356

[244] E. Knuplez and G. Marsche , “An Updated Review of Pro‐ and Anti‐Inflammatory Properties of Plasma Lysophosphatidylcholines in the Vascular System,” International Journal of Molecular Sciences 21 (2020): 4501. 32599910 10.3390/ijms21124501 PMC7350010

[245] Q. Zhang , W. Zhang , J. Liu , et al., “Lysophosphatidylcholine Promotes Intercellular Adhesion Molecule‐1 and Vascular Cell Adhesion Molecule‐1 Expression in Human Umbilical Vein Endothelial Cells via an Orphan G Protein Receptor 2‐Mediated Signaling Pathway,” Bioengineered 12 (2021): 4520–4535. 34346841 10.1080/21655979.2021.1956671 PMC8806654

[246] F. Coutant , L. Perrin‐Cocon , S. Agaugué , T. Delair , P. André , and V. Lotteau , “Mature Dendritic Cell Generation Promoted by Lysophosphatidylcholine,” Journal of Immunology 169 (2002): 1688–1695. 10.4049/jimmunol.169.4.1688 12165488

[247] J. H. Kabarowski , “G2A and LPC: Regulatory Functions in Immunity,” Prostaglandins & Other Lipid Mediators 89 (2009): 73–81. 19383550 10.1016/j.prostaglandins.2009.04.007 PMC2740801

[248] R. Corrêa , L. F. Silva , D. J. Ribeiro , et al., “Lysophosphatidylcholine Induces NLRP3 Inflammasome‐Mediated Foam Cell Formation and Pyroptosis in Human Monocytes and Endothelial Cells,” Frontiers in Immunology 10 (2019): 2927. 31998284 10.3389/fimmu.2019.02927 PMC6962110

[249] S. Jiang , D. W. Park , W. S. Stigler , et al., “Mitochondria and AMP‐Activated Protein Kinase‐Dependent Mechanism of Efferocytosis,” Journal of Biological Chemistry 288 (2013): 26013–26026. 23897815 10.1074/jbc.M113.489468 PMC3764806

[250] S. Casagrande , G. B. Sopetto , G. Bertalot , et al., “Immune‐Related Adverse Events due to Cancer Immunotherapy: Immune Mechanisms and Clinical Manifestations,” Cancers (Basel) 16 (2024): 1440. 38611115 10.3390/cancers16071440 PMC11011060

[251] J. Choi and S. Y. Lee , “Clinical Characteristics and Treatment of Immune‐Related Adverse Events of Immune Checkpoint Inhibitors,” Immune Network 20 (2020): e9. 32158597 10.4110/in.2020.20.e9 PMC7049586

[252] N. Guezour , G. Soussi , S. Brosseau , et al., “Grade 3‐4 Immune‐Related Adverse Events Induced by Immune Checkpoint Inhibitors in Non‐Small‐Cell Lung Cancer (NSCLC) Patients Are Correlated With Better Outcome: A Real‐Life Observational Study,” Cancers (Basel) 14 (2022): 3878. 36010872 10.3390/cancers14163878 PMC9405595

[253] D. Y. Wang , J. E. Salem , J. V. Cohen , et al., “Fatal Toxic Effects Associated With Immune Checkpoint Inhibitors: A Systematic Review and Meta‐Analysis,” JAMA Oncology 4 (2018): 1721–1728. 30242316 10.1001/jamaoncol.2018.3923 PMC6440712

[254] P. Saminathan , A. Gibbons , I. Mathews , et al., “LPC 18:2‐Driven Apoptosis in Neutrophils Is Non‐Inflammatory and Lipid Raft Dependent,” preprint, bioRxivorg (2025), 10.64898/2025.12.09.693266.

[255] H. C. Tews , T. Elger , M. Huss , et al., “Decline in Serum Lysophosphatidylcholine Species in Patients With Severe Inflammatory Bowel Disease,” Journal of Clinical Medicine 14 (2025): 5485. 40807102 10.3390/jcm14155485 PMC12347743

[256] K. Paapstel , J. Kals , J. Eha , et al., “Inverse Relations of Serum Phosphatidylcholines and Lysophosphatidylcholines With Vascular Damage and Heart Rate in Patients With Atherosclerosis,” Nutrition, Metabolism, and Cardiovascular Diseases 28 (2018): 44–52. 10.1016/j.numecd.2017.07.011 28986077

[257] S. H. Tan , H. W. L. Koh , J. Y. Chua , et al., “Variability of the Plasma Lipidome and Subclinical Coronary Atherosclerosis,” Arteriosclerosis, Thrombosis, and Vascular Biology 42 (2022): 100–112. 34809445 10.1161/ATVBAHA.121.316847 PMC8691371

[258] P. J. Meikle , G. Wong , D. Tsorotes , et al., “Plasma Lipidomic Analysis of Stable and Unstable Coronary Artery Disease,” Arteriosclerosis, Thrombosis, and Vascular Biology 31 (2011): 2723–2732. 21903946 10.1161/ATVBAHA.111.234096

[259] W. Drobnik , G. Liebisch , F. X. Audebert , et al., “Plasma Ceramide and Lysophosphatidylcholine Inversely Correlate With Mortality in Sepsis Patients,” Journal of Lipid Research 44 (2003): 754–761. 12562829 10.1194/jlr.M200401-JLR200

[260] A. Montagne , S. R. Barnes , M. D. Sweeney , et al., “Blood‐Brain Barrier Breakdown in the Aging Human Hippocampus,” Neuron 85 (2015): 296–302. 25611508 10.1016/j.neuron.2014.12.032 PMC4350773

[261] H. Chew , V. A. Solomon , and A. N. Fonteh , “Involvement of Lipids in Alzheimer's Disease Pathology and Potential Therapies,” Frontiers in Physiology 11 (2020): 598. 32581851 10.3389/fphys.2020.00598 PMC7296164

[262] J. W. Kinney , S. M. Bemiller , A. S. Murtishaw , A. M. Leisgang , A. M. Salazar , and B. T. Lamb , “Inflammation as a Central Mechanism in Alzheimer's Disease,” Alzheimer's & Dementia: Translational Research & Clinical Interventions 4 (2018): 575–590. 10.1016/j.trci.2018.06.014 PMC6214864 30406177

[263] S.‐W. Xu , I. Ilyas , and J.‐P. Weng , “Endothelial Dysfunction in COVID‐19: An Overview of Evidence, Biomarkers, Mechanisms and Potential Therapies,” Acta Pharmacologica Sinica 44 (2023): 695–709. 36253560 10.1038/s41401-022-00998-0 PMC9574180

[264] A. Rezaei , S. Neshat , and K. Heshmat‐Ghahdarijani , “Alterations of Lipid Profile in COVID‐19: A Narrative Review,” Current Problems in Cardiology 47 (2022): 100907. 34272088 10.1016/j.cpcardiol.2021.100907 PMC8161768

[265] M. C. Pelle , I. Zaffina , S. Lucà , et al., “Endothelial Dysfunction in COVID‐19: Potential Mechanisms and Possible Therapeutic Options,” Life (Basel) 12 (2022): 1605. 36295042 10.3390/life12101605 PMC9604693

[266] K. Lin , J. Cai , J. Guo , et al., “Multi‐Omics Landscapes Reveal Heterogeneity in Long COVID Patients Characterized With Enhanced Neutrophil Activity,” Journal of Translational Medicine 22 (2024): 753. 39135185 10.1186/s12967-024-05560-6 PMC11318262

[267] T. Molnar , A. Lehoczki , M. Fekete , et al., “Mitochondrial Dysfunction in Long COVID: Mechanisms, Consequences, and Potential Therapeutic Approaches,” GeroScience 46 (2024): 5267–5286. 38668888 10.1007/s11357-024-01165-5 PMC11336094

[268] E. Casas‐Fernández , C. Peña‐Bautista , M. Baquero , and C. Cháfer‐Pericás , “Lipids as Early and Minimally Invasive Biomarkers for Alzheimer's Disease,” Current Neuropharmacology 20 (2022): 1613–1631. 34727857 10.2174/1570159X19666211102150955 PMC9881089

[269] P. M. Kris‐Etherton , W. S. Harris , L. J. Appel , and American Heart Association. Nutrition Committee , “Fish Consumption, Fish Oil, Omega‐3 Fatty Acids, and Cardiovascular Disease,” Circulation 106 (2002): 2747–2757. 12438303 10.1161/01.cir.0000038493.65177.94

[270] J. Thomas , C. J. Thomas , J. Radcliffe , and C. Itsiopoulos , “Omega‐3 Fatty Acids in Early Prevention of Inflammatory Neurodegenerative Disease: A Focus on Alzheimer's Disease,” BioMed Research International 2015 (2015): 172801. 26301243 10.1155/2015/172801 PMC4537710

[271] R. Avallone , G. Vitale , and M. Bertolotti , “Omega‐3 Fatty Acids and Neurodegenerative Diseases: New Evidence in Clinical Trials,” International Journal of Molecular Sciences 20 (2019): 4256. 31480294 10.3390/ijms20174256 PMC6747747

[272] B. H. Wong and D. L. Silver , “Mfsd2a: A Physiologically Important Lysolipid Transporter in the Brain and Eye,” Advances in Experimental Medicine and Biology 1276 (2020): 223–234. 32705603 10.1007/978-981-15-6082-8_14

[273] P. C. R. Yalagala , D. Sugasini , S. Dasarathi , K. Pahan , and P. V. Subbaiah , “Dietary Lysophosphatidylcholine‐EPA Enriches Both EPA and DHA in the Brain: Potential Treatment for Depression,” Journal of Lipid Research 60 (2019): 566–578. 30530735 10.1194/jlr.M090464 PMC6399499

[274] D. Sugasini , R. Thomas , P. C. R. Yalagala , L. M. Tai , and P. V. Subbaiah , “Dietary Docosahexaenoic Acid (DHA) as Lysophosphatidylcholine, but Not as Free Acid, Enriches Brain DHA and Improves Memory in Adult Mice,” Scientific Reports 7 (2017): 11263. 28900242 10.1038/s41598-017-11766-0 PMC5596017

[275] E. J. Baker , E. A. Miles , G. C. Burdge , P. Yaqoob , and P. C. Calder , “Metabolism and Functional Effects of Plant‐Derived Omega‐3 Fatty Acids in Humans,” Progress in Lipid Research 64 (2016): 30–56. 27496755 10.1016/j.plipres.2016.07.002

[276] V. Alakbarzade , A. Hameed , D. Q. Quek , et al., “A Partially Inactivating Mutation in the Sodium‐Dependent Lysophosphatidylcholine Transporter MFSD2A Causes a Non‐Lethal Microcephaly Syndrome,” Nature Genetics 47 (2015): 814–817. 26005865 10.1038/ng.3313

[277] A. Guemez‐Gamboa , L. N. Nguyen , H. Yang , et al., “Inactivating Mutations in MFSD2A, Required for Omega‐3 Fatty Acid Transport in Brain, Cause a Lethal Microcephaly Syndrome,” Nature Genetics 47 (2015): 809–813. 26005868 10.1038/ng.3311 PMC4547531

[278] J. P. Chan , B. H. Wong , C. F. Chin , et al., “The Lysolipid Transporter Mfsd2a Regulates Lipogenesis in the Developing Brain,” PLoS Biology 16 (2018): e2006443. 30074985 10.1371/journal.pbio.2006443 PMC6093704

[279] L. N. Nguyen , D. Ma , G. Shui , et al., “Mfsd2a Is a Transporter for the Essential Omega‐3 Fatty Acid Docosahexaenoic Acid,” Nature 509 (2014): 503–506. 24828044 10.1038/nature13241

[280] S. C. Dyall , “Long‐Chain Omega‐3 Fatty Acids and the Brain: A Review of the Independent and Shared Effects of EPA, DPA and DHA,” Frontiers in Aging Neuroscience 7 (2015): 52. 25954194 10.3389/fnagi.2015.00052 PMC4404917

[281] T. Greenhalgh , M. Sivan , A. Perlowski , and J. Ž. Nikolich , “Long COVID: A Clinical Update,” Lancet 404 (2024): 707–724. 39096925 10.1016/S0140-6736(24)01136-X

[282] L. L. O'Mahoney , A. Routen , C. Gillies , et al., “The Prevalence and Long‐Term Health Effects of Long Covid Among Hospitalised and Non‐Hospitalised Populations: A Systematic Review and Meta‐Analysis,” EClinicalMedicine 55 (2023): 101762. 36474804 10.1016/j.eclinm.2022.101762 PMC9714474

[283] H. E. Davis , L. McCorkell , J. M. Vogel , and E. J. Topol , “Long COVID: Major Findings, Mechanisms and Recommendations,” Nature Reviews Microbiology 21 (2023): 133–146. 36639608 10.1038/s41579-022-00846-2 PMC9839201

[284] M. C. Basil and B. D. Levy , “Specialized Pro‐Resolving Mediators: Endogenous Regulators of Infection and Inflammation,” Nature Reviews. Immunology 16 (2016): 51–67. 10.1038/nri.2015.4 PMC5242505 26688348

[285] J. Ponce , A. Ulu , C. Hanson , et al., “Role of Specialized Pro‐Resolving Mediators in Reducing Neuroinflammation in Neurodegenerative Disorders,” Frontiers in Aging Neuroscience 14 (2022): 780811. 35250536 10.3389/fnagi.2022.780811 PMC8891627

[286] R. F. Damiano , C. C. d. A. Rocca , A. d. P. Serafim , et al., “Cognitive Impairment in Long‐COVID and Its Association With Persistent Dysregulation in Inflammatory Markers,” Frontiers in Immunology 14 (2023): 1174020. 37287969 10.3389/fimmu.2023.1174020 PMC10242059

[287] N. Kappelmann , R. Dantzer , and G. M. Khandaker , “Interleukin‐6 as Potential Mediator of Long‐Term Neuropsychiatric Symptoms of COVID‐19,” Psychoneuroendocrinology 131 (2021): 105295. 34119855 10.1016/j.psyneuen.2021.105295 PMC8172271

[288] G. Kenny , L. Townsend , S. Savinelli , and P. W. G. Mallon , “Long COVID: Clinical Characteristics, Proposed Pathogenesis and Potential Therapeutic Targets,” Frontiers in Molecular Biosciences 10 (2023): 1157651. 37179568 10.3389/fmolb.2023.1157651 PMC10171433

[289] D. Li , J. R. Misialek , E. Boerwinkle , et al., “Plasma Phospholipids and Prevalence of Mild Cognitive Impairment and/or Dementia in the ARIC Neurocognitive Study (ARIC‐NCS),” Alzheimer's & dementia (Amsterdam, Netherlands) 3 (2016): 73–82. 10.1016/j.dadm.2016.02.008 PMC4925799 27408938

[290] Q. He , Q. Li , J. Zhao , et al., “Relationship Between Plasma Lipids and Mild Cognitive Impairment in the Elderly Chinese: A Case‐Control Study,” Lipids in Health and Disease 15 (2016): 146. 27595570 10.1186/s12944-016-0320-6 PMC5011904

[291] M. van Oijen , F. J. de Jong , J. C. Witteman , A. Hofman , P. J. Koudstaal , and M. M. Breteler , “Atherosclerosis and Risk for Dementia,” Annals of Neurology 61 (2007): 403–410. 17328068 10.1002/ana.21073

[292] J. P. Ferrari‐Souza , B. Bellaver , P. C. L. Ferreira , et al., “APOEε4 Potentiates Amyloid β Effects on Longitudinal Tau Pathology,” Nature Aging 3 (2023): 1210–1218. 37749258 10.1038/s43587-023-00490-2 PMC10592050

[293] M. S. Baek , H. Cho , H. S. Lee , J. H. Lee , Y. H. Ryu , and C. H. Lyoo , “Effect of APOE ε4 Genotype on Amyloid‐β and Tau Accumulation in Alzheimer's Disease,” Alzheimer's Research & Therapy 12 (2020): 140. 10.1186/s13195-020-00710-6 PMC7603688 33129364

[294] J. Qian , R. A. Betensky , B. T. Hyman , and A. Serrano‐Pozo , “Association of APOE Genotype With Heterogeneity of Cognitive Decline Rate in Alzheimer Disease,” Neurology 96 (2021): e2414–e2428. 33771840 10.1212/WNL.0000000000011883 PMC8166439

[295] P. Saminathan , S. McArdle , M. Corey , et al., “Unmasking Early Microglial Remodeling in an Alzheimer's Disease Mouse Model,” Frontiers in Cellular Neuroscience 19 (2025): 1720382. 41550298 10.3389/fncel.2025.1720382 PMC12807895

[296] K. R. Sadleir , W. A. Eimer , S. L. Cole , and R. Vassar , “Aβ Reduction in BACE1 Heterozygous Null 5XFAD Mice Is Associated With Transgenic APP Level,” Molecular Neurodegeneration 10 (2015): 1. 25567526 10.1186/1750-1326-10-1 PMC4297413

[297] Z. Jin , Y. Lu , H. Tang , and H. Cui , “Integrating Neuroinflammation Biomarkers Into the ATN(X) Framework: Advances in Alzheimer's Pathogenesis, Diagnosis, and Insights From Non‐Human Primate Models,” Alzheimer's & Dementia 21 (2025): e70472. 10.1002/alz.70472 PMC12344583 40801241

[298] R. Rajmohan and P. H. Reddy , “Amyloid‐Beta and Phosphorylated Tau Accumulations Cause Abnormalities at Synapses of Alzheimer's Disease Neurons,” Journal of Alzheimer's Disease 57 (2017): 975–999. 10.3233/JAD-160612 PMC5793225 27567878

[299] S. Lavisse , D. García‐Lorenzo , M.‐A. Peyronneau , et al., “Optimized Quantification of Translocator Protein Radioligand ^18^F‐DPA‐714 Uptake in the Brain of Genotyped Healthy Volunteers,” Journal of Nuclear Medicine 56 (2015): 1048–1054. 26025960 10.2967/jnumed.115.156083

[300] S. E. Watling , T. Gill , E. V. Gaudette , et al., “Investigating TSPO Levels in Occupation‐Related Posttraumatic Stress Disorder,” Scientific Reports 13 (2023): 4970. 36973385 10.1038/s41598-023-31327-y PMC10041517

[301] M. L. Vanden Noven , M. Anselmo , C. T. Tahsin , J. R. Carter , and M. L. Keller‐Ross , “A Review of the Historical Use of Sex as a Biological Variable in the American Journal of Physiology‐Heart and Circulatory Physiology,” American Journal of Physiology. Heart and Circulatory Physiology 325 (2023): H768–H773. 37594486 10.1152/ajpheart.00278.2023 PMC10643001

[302] A. K. Beery and I. Zucker , “Sex Bias in Neuroscience and Biomedical Research,” Neuroscience and Biobehavioral Reviews 35 (2011): 565–572. 20620164 10.1016/j.neubiorev.2010.07.002 PMC3008499

[303] R. M. Shansky and C. S. Woolley , “Considering Sex as a Biological Variable Will Be Valuable for Neuroscience Research,” Journal of Neuroscience 36 (2016): 11817–11822. 27881768 10.1523/JNEUROSCI.1390-16.2016 PMC5125240

[304] I. Zucker , “The Mixed Legacy of the Rat Estrous Cycle,” Biology of Sex Differences 14 (2023): 55. 37667337 10.1186/s13293-023-00542-7 PMC10476291

[305] Institute of Medicine (US) Committee on the Ethical and Legal Issues Relating to the Inclusion of Women in Clinical Studies , Women and Health Research: Ethical and Legal Issues of Including Women in Clinical Studies: Volume 2: Workshop and Commissioned Papers (National Academies Press (US), 1999), 10.17226/2343. 25144106

[306] J. A. Clayton and F. S. Collins , “Policy: NIH to Balance Sex in Cell and Animal Studies,” Nature 509 (2014): 282–283. 24834516 10.1038/509282a PMC5101948

[307] M. E. Arnegard , L. A. Whitten , C. Hunter , and J. A. Clayton , “Sex as a Biological Variable: A 5‐Year Progress Report and Call to Action,” Journal of Women's Health (2002) 29 (2020): 858–864. 10.1089/jwh.2019.8247 PMC7476377 31971851

[308] M. H. Barlek , J. R. Rouan , T. G. Wyatt , I. Helenowski , and M. R. Kibbe , “The Persistence of Sex Bias in High‐Impact Clinical Research,” Journal of Surgical Research 278 (2022): 364–374. 35687931 10.1016/j.jss.2022.04.077 PMC11953574

[309] G. M. Mamlouk , D. M. Dorris , L. R. Barrett , and J. Meitzen , “Sex Bias and Omission in Neuroscience Research Is Influenced by Research Model and Journal, but Not Reported NIH Funding,” Frontiers in Neuroendocrinology 57 (2020): 100835. 32070715 10.1016/j.yfrne.2020.100835 PMC7225067

[310] D. M. Conde , R. C. Verdade , A. L. R. Valadares , L. F. B. Mella , A. O. Pedro , and L. Costa‐Paiva , “Menopause and Cognitive Impairment: A Narrative Review of Current Knowledge,” World Journal of Psychiatry 11 (2021): 412–428. 34513605 10.5498/wjp.v11.i8.412 PMC8394691

[311] M. McCarthy and A. P. Raval , “The Peri‐Menopause in a Woman's Life: A Systemic Inflammatory Phase That Enables Later Neurodegenerative Disease,” Journal of Neuroinflammation 17 (2020): 317. 33097048 10.1186/s12974-020-01998-9 PMC7585188

[312] A. Christensen and C. J. Pike , “Menopause, Obesity and Inflammation: Interactive Risk Factors for Alzheimer's Disease,” Frontiers in Aging Neuroscience 7 (2015): 130. 26217222 10.3389/fnagi.2015.00130 PMC4493396

[313] S. J. Teipel , Y. Tang , and A. Khachaturian , “Sex Differences in Treatment Effects of Lecanemab and Donanemab: A Bayesian Reanalysis of CLARITY‐AD and TRAILBLAZER‐ALZ2,” Alzheimer's & Dementia (New York, N. Y.) 11 (2025): e70155. 10.1002/trc2.70155 PMC12412750 40918062

[314] D. Andrews , S. Ducharme , H. Chertkow , M. P. Sormani , and D. L. Collins , “The Higher Benefit of Lecanemab in Males Compared to Females in CLARITY AD Is Probably due to a Real Sex Effect,” Alzheimer's & Dementia 21 (2025): e14467. 10.1002/alz.14467 PMC11779744 39887549

[315] J. Mestas and C. C. W. Hughes , “Of Mice and Not Men: Differences Between Mouse and Human Immunology,” Journal of Immunology (Baltimore, Md.: 1950) 172 (2004): 2731–2738. 14978070 10.4049/jimmunol.172.5.2731

[316] M. Bailey , Z. Christoforidou , and M. C. Lewis , “The Evolutionary Basis for Differences Between the Immune Systems of Man, Mouse, Pig and Ruminants,” Veterinary Immunology and Immunopathology 152 (2013): 13–19. 23078904 10.1016/j.vetimm.2012.09.022

[317] K. N. Z. Fuller and J. P. Thyfault , “Barriers in Translating Preclinical Rodent Exercise Metabolism Findings to Human Health,” Journal of Applied Physiology (1985) 130 (2021): 182–192. 10.1152/japplphysiol.00683.2020 PMC7944931 33180643

[318] A. M. Sabogal‐Guáqueta , A. Marmolejo‐Garza , M. Trombetta‐Lima , et al., “Species‐Specific Metabolic Reprogramming in Human and Mouse Microglia During Inflammatory Pathway Induction,” Nature Communications 14 (2023): 6454. 10.1038/s41467-023-42096-7 PMC10575978 37833292

[319] S. V. Koebele and H. A. Bimonte‐Nelson , “Modeling Menopause: The Utility of Rodents in Translational Behavioral Endocrinology Research,” Maturitas 87 (2016): 5–17. 27013283 10.1016/j.maturitas.2016.01.015 PMC4829404

[320] S. P. Ward , “Thalidomide and Congenital Abnormalities,” BMJ (Clinical Research Ed.) 2 (1962): 646–647. 10.1136/bmj.2.5305.646 PMC1925989 14004945

[321] S. M. Neuner , S. E. Heuer , M. J. Huentelman , K. M. S. O'Connell , and C. C. Kaczorowski , “Harnessing Genetic Complexity to Enhance Translatability of Alzheimer's Disease Mouse Models: A Path Toward Precision Medicine,” Neuron 101 (2019): 399–411.e5. 30595332 10.1016/j.neuron.2018.11.040 PMC6886697

[322] K. M. S. O'Connell , A. R. Ouellette , S. M. Neuner , A. R. Dunn , and C. C. Kaczorowski , “Genetic Background Modifies CNS‐Mediated Sensorimotor Decline in the AD‐BXD Mouse Model of Genetic Diversity in Alzheimer's Disease,” Genes, Brain and Behavior 18 (2019): e12603. 31381246 10.1111/gbb.12603 PMC6899779

[323] A. Silveira Prudente , S. Hoon Lee , J. Roh , et al., “Microglial STING Activation Alleviates Nerve Injury‐Induced Neuropathic Pain in Male but Not Female Mice,” Brain, Behavior, and Immunity 117 (2024): 51–65. 38190983 10.1016/j.bbi.2024.01.003 PMC11034751

